# A Complex Structural Variation on Chromosome 27 Leads to the Ectopic Expression of *HOXB8* and the Muffs and Beard Phenotype in Chickens

**DOI:** 10.1371/journal.pgen.1006071

**Published:** 2016-06-02

**Authors:** Ying Guo, Xiaorong Gu, Zheya Sheng, Yanqiang Wang, Chenglong Luo, Ranran Liu, Hao Qu, Dingming Shu, Jie Wen, Richard P. M. A. Crooijmans, Örjan Carlborg, Yiqiang Zhao, Xiaoxiang Hu, Ning Li

**Affiliations:** 1 State Key Laboratory for Agro-Biotechnology, China Agricultural University, Beijing, China; 2 National Engineering Laboratory for Animal Breeding, China Agricultural University, Beijing, China; 3 Division of Computational Genetics, Department of Clinical Sciences, Swedish University of Agricultural Sciences, Uppsala, Sweden; 4 Institute of Animal Science, Guangdong Academy of Agricultural Sciences, Guangzhou, Guangdong, China; 5 Institute of Animal Science, Chinese Academy of Agricultural Sciences, Beijing, China; 6 Animal Breeding and Genomics Centre, Wageningen University, Wageningen, the Netherlands; University of Bern, SWITZERLAND

## Abstract

Muffs and beard (Mb) is a phenotype in chickens where groups of elongated feathers gather from both sides of the face (muffs) and below the beak (beard). It is an autosomal, incomplete dominant phenotype encoded by the *Muffs and beard* (*Mb*) locus. Here we use genome-wide association (GWA) analysis, linkage analysis, Identity-by-Descent (IBD) mapping, array-CGH, genome re-sequencing and expression analysis to show that the *Mb* allele causing the Mb phenotype is a derived allele where a complex structural variation (SV) on GGA27 leads to an altered expression of the gene *HOXB8*. This *Mb* allele was shown to be completely associated with the Mb phenotype in nine other independent Mb chicken breeds. The *Mb* allele differs from the wild-type *mb* allele by three duplications, one in tandem and two that are translocated to that of the tandem repeat around 1.70 Mb on GGA27. The duplications contain total seven annotated genes and their expression was tested during distinct stages of Mb morphogenesis. A continuous high ectopic expression of *HOXB8* was found in the facial skin of Mb chickens, strongly suggesting that *HOXB8* directs this regional feather-development. In conclusion, our results provide an interesting example of how genomic structural rearrangements alter the regulation of genes leading to novel phenotypes. Further, it again illustrates the value of utilizing derived phenotypes in domestic animals to dissect the genetic basis of developmental traits, herein providing novel insights into the likely role of *HOXB8* in feather development and differentiation.

## Author Summary

Genetic variation is a key part for the study of evolution, development and differentiation. In domestic animals, many breeds display striking phenotypes that differentiate them from their wild ancestors. Several of these have been related to structural variations, including *Fibromelanosis* and *Rose-comb* in chickens, *Double-muscled* and *Osteopetrosis* in cattle, *Cone degeneration* in dogs, and *White coat color* in pigs. The feather is a type of skin appendages that exists in multiple variants on different body parts, and the derived feathering phenotypes in domestic birds are perfect resources to decipher the mechanisms regulating feather development and differentiation. Here we study the genetics of the Muffs and beard trait, a variant that alters the feather development in the facial area of chickens. We show that this phenotype is associated with a genomic structural variant that leads to an ectopic expression of *HOXB8* in the facial skin during feather development. This is thus another example of how structural variants in the genome lead to novel, derived phenotypic changes in domestic animals and suggests an important role for *HOXB8* in feather development.

## Introduction

For several thousand years, domesticated animals have been subjected to a combination of natural and artificial selection, and during this process accumulated numerous phenotypic variations. The resulting genetic and phenotypic diversity in domesticated animals provides an excellent basis for improving our understanding of the role of genes in development and disease resistance. The feather, a complex integumentary appendage, is a characteristic feature critical for avian functions such as flight, communication, waterproofing, and thermoregulation. It consists of vanes, rachises, barbs, afterfeathers and calami, all of which may vary in number, size, and shape. Such variations contribute to the diversiform branching patterns of various types of feathers [1]. Due to the structural diversity and its hierarchical development, the feather has been an important topic of research in developmental biology and genetic research.

The chicken is an attractive avian species for genetics research, due to both the abundant genetic and phenotypic diversity as well as the available genomic resources. A few key characteristics of chicken, including its vast economic importance in food production, human-like diseases, and manipulability of the embryos, have facilitated research into topics of importance for agriculture, medicine, and fundamental biology [2]. After domestication, spontaneous mutations in chickens have led to various phenotypic variations, such as *Crest* (*Cr*) [3], *Naked neck* (*Nc*) [4], *Scaleless* (*Sc*) [5], *Frizzle* (*Fr)* [6], *Silky* (*H*) [7], *Ear tufts* (*Et*) [8], *Ptilopody or feathered shank* (*Pti*) [9], *Vulture hocks* (*Vh*) [9,10], and *Muffs and beard* (*Mb*) [11]. Until now, several mutations underlying these phenotypic variations have been identified. From the variations with known genetic causes, it is apparent that small genetic changes often lead to striking phenotypic differences. The variants for which the underlying genetic mechanisms are still unknown thus provide excellent models in a powerful way to study the fundamental principles of feather development and differentiation.

In domestic animals, recent studies have identified that an increasing number of structural variations (SVs) are found to be associated with phenotypic changes. An interesting typical example in the chicken is the dissection of the molecular architectures underlying three comb variants: *Rose-comb*, *Pea-comb*, and *Duplex-comb*, where all were found to be controlled by SVs in the genome, rather than single nucleotide changes. *Rose-comb* is caused by a large inversion that induces the transient ectopic expression of *MNR2* [12]. *Pea-comb* results from a massive-amplification event in intron 1 of *SOX5* leading to its ectopic expression in mesenchymal cells and, therefore, form the special comb [13]. The *Duplex-comb* mutation is a tandem duplication containing several conserved putative regulatory elements located upstream of *EOMES* and changes its expression [14]. All these suggested the SV as a primary contributor to phenotypic diversities and developmental defects.

**Fig 1:**
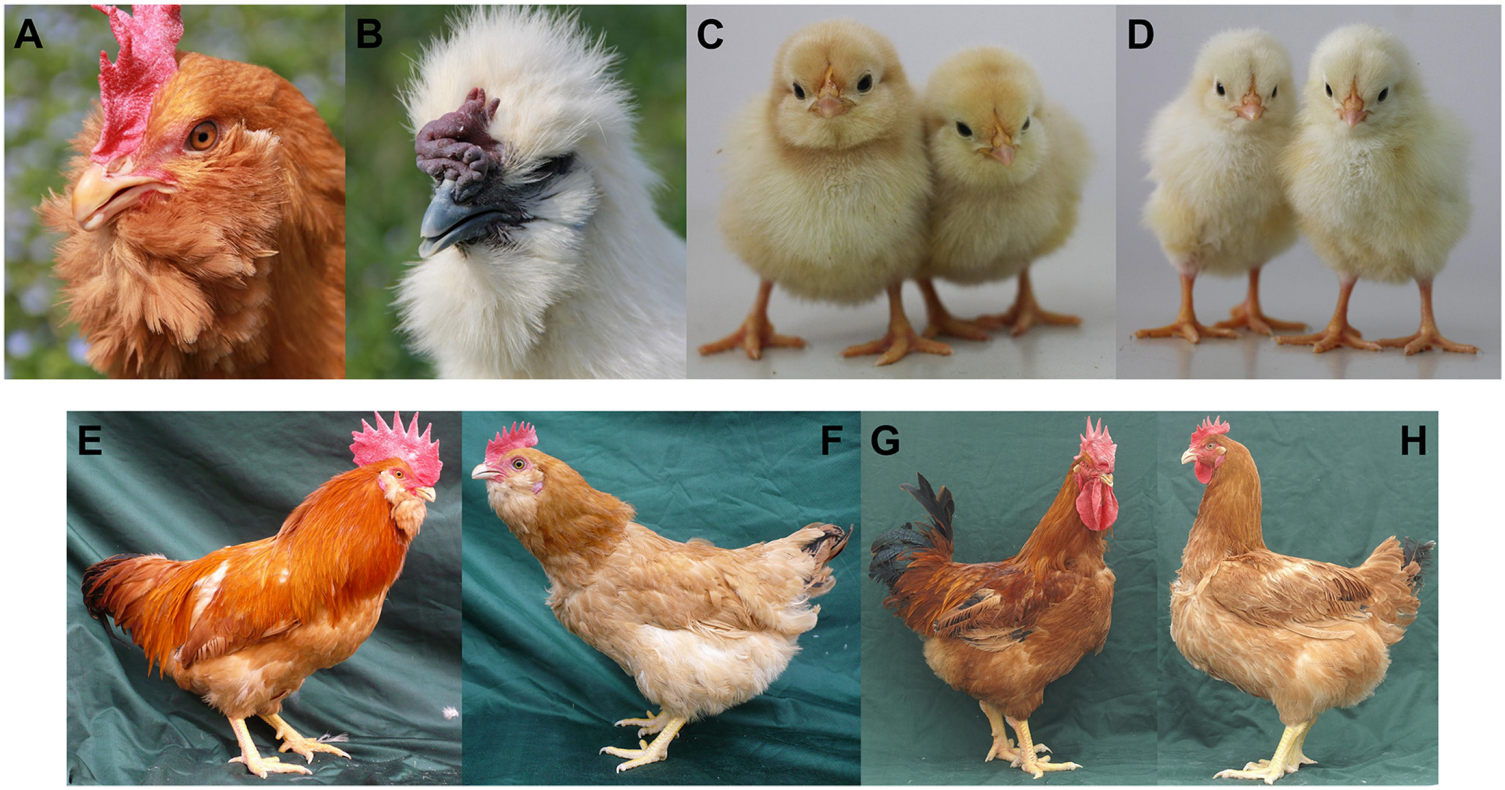
Muffs and beard phenotype in Chinese domestic chickens. (A) Huiyang Bearded (HB) chicken. (B) Silky-feather chicken with Muffs and beard. (C) Newly hatched Mb chicks from the HB broiler breed. (D) Newly hatched wild-type chicks from White Leghorn breed. Male (E) and female (F) Huiyang Bearded chickens that were founders of the HB & HQLA family. Male (G) and female (H) birds from the High quality chicken Line A were also founders of HB & HQLA family.

This study explores the genetics of the Muffs and beard phenotype (Mb) (Fig 1) in chickens, which consist of tufts of elongated feathers projecting around the face and the beak [11]. Although it has earlier been confirmed to be a single-locus, autosomal, incompletely dominant trait [15,16], the causal mutation still remains unknown. There are several breeds of chickens displaying this special feathering phenotype around the world, including Huiyang Bearded, Silkie, Beijing-You, Xiangdong, Piao, Dutch Polish Bantam, Dutch owl, Dutch owl bantam, Brabanter, Brabanter bantam, and others. Here we show that the *Mb* allele that causes the Mb phenotype in chickens is a structural mutation resulting from duplications of three regions on chicken chromosome 27 (GGA27), where two of these have also been translocated and inserted between the tandem repeats of the first duplication. We also demonstrate that this structural rearrangement leads to an altered ectopic expression of *HOXB8* in the facial skin, making it a highly likely candidate mechanism for the presence of the elongated feathers characterizing the Mb phenotype.

## Results

### Mapping and fine-mapping of the *Mb* locus to a 48-kb region on GGA27

**Fig 2:**
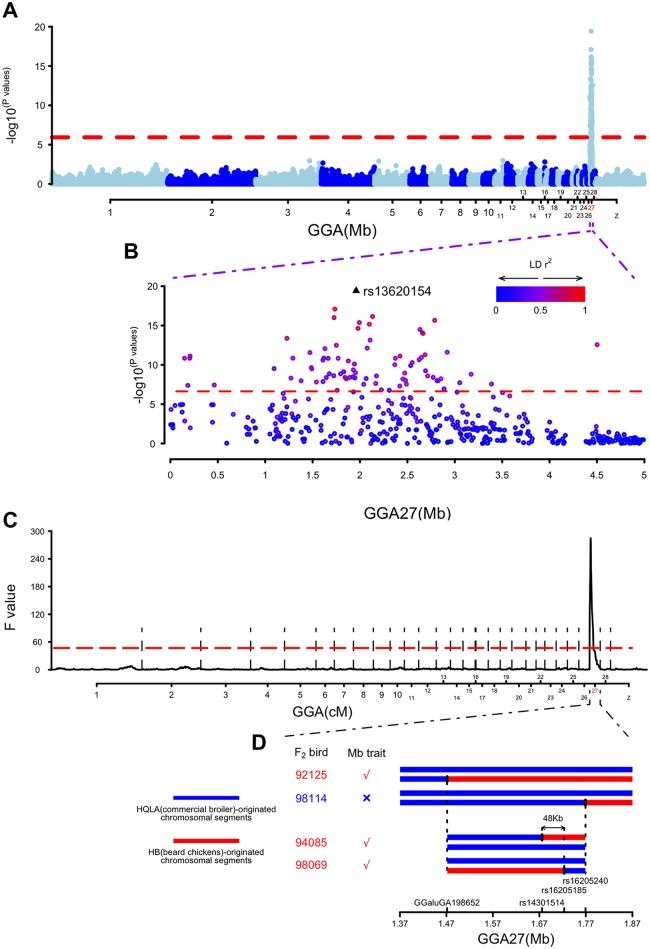
Results from the Genome-Wide Association, linkage and shared IBD analyses on the Muffs and beard (Mb) trait in HB × HQLA population. (A) The Manhattan plot from the Genome-wide association analysis for the Mb phenotype at 10 weeks of age. X-axis shows the physical positions in Mb for each marker along the chromosomes, and the y-axis shows -log_10_ *p* values for the association tests. (B) A scatter plot illustrating all SNPs tested on GGA27 for the Mb trait at 10 weeks of age. The peak SNP (rs13620154 on GGA27 at 1,959,687 bp) is marked with a filled triangle, while other SNPs are marked with dots. The colors of the dots indicate their LD (r^2^) with the peak SNP. (C) The whole-genome linkage analysis for the Mb trait at 10 weeks of age. The x-axis shows the genetic positions in centiMorgan (cM) along chromosomes, and the y-axis shows the F values for each position. Vertical dashed lines are used to distinguish the chromosomes. (D) Shared IBD analysis is shown in schematic form in this plot. Each bar represents the *Mb* locus identified in linkage analysis for one F_2_ bird. Bars in red refers to chromosomal segments originated from line HB, bars in blue refers to segments originated from line HQLA. Four breakpoints of recombination were indicated by the corresponding SNP names on the x-axis. The dashed lines indicated the boundary defined by corresponding recombinant individuals. The arrows pointed out the location of the final fine-mapped 48-kb interval between two SNPs (rs14301514 and rs16205185).

An F_2_ intercross population between Huiyang Bearded chickens (HB, a Chinese native Mb breed) and High Quality of Chicken Line A (HQLA, a non-Mb broiler line) consisting of 585 birds were bred as our primary mapping population [17]. By testing for associations between the genotypes from a 60k SNP chip and the Mb phenotype at 10 weeks of age in this population, we identified a single, highly significantly (p<10^−19^; Fig 2) associated 2.5 Mb (1.1 Mb to 3.5 Mb) region on GGA27. We then used a second population established using a Chinese local breed, Beijing-You chickens [18], which had Mb trait segregating in the population, to perform a validating association study. Its result further confirmed that the *Mb* locus was located in the first half of GGA27 (S1 Fig and S1 Table).

A linkage analysis across GGA27, that utilized the HB × HQLA pedigree and the 60k marker information to assign line-origin probabilities every cM across the chromosome and test for associations between HB- and HQLA-derived chromosomes, identified a peak signal at the nearest tested location on the chromosome (5 cM; 1.61 Mb; Fig 2C) to that of the association study (Fig 2A and 2B).

Under the assumption that a single bi-allelic locus caused the phenotype, we also applied an IBD (Identity by Descent) mapping approach to fine map the *Mb* locus. By searching the minimal chromosomal segments that were shared among all Mb F_2_ chickens, we acquired a 48-kb region that spans six SNPs for the *Mb* locus (1.68–1.72 Mb; Fig 2D). Together these results provided a high-resolution mapping of the *Mb* locus to guide the following search for the functional mutation causing the Mb trait.

### A complex structural variation is detected in the *Mb* locus on GGA27

**Fig 3:**
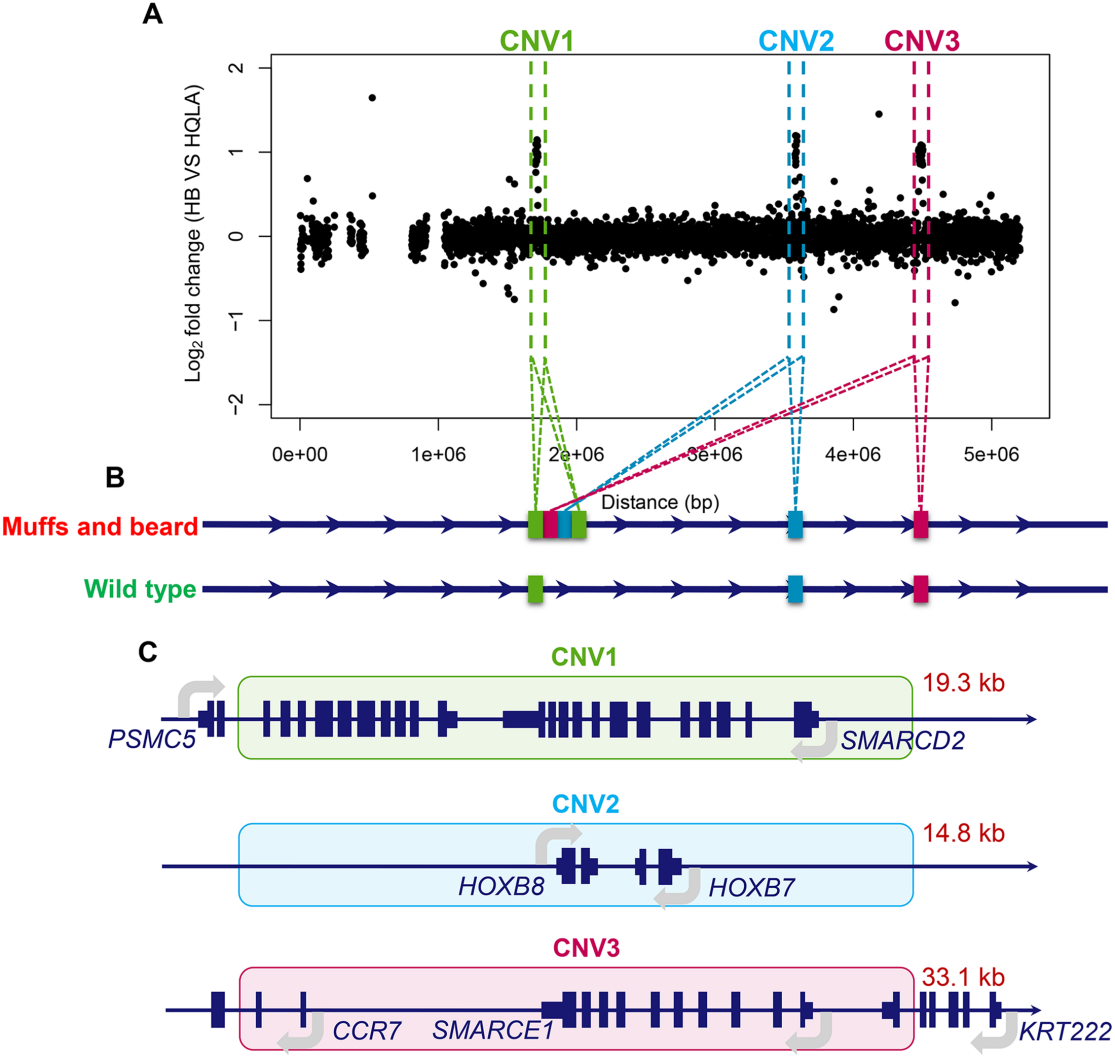
Illustration of the read depth analysis that confirmed the copy number variations on GGA27 and the following fine-mapping of their structural rearrangements. (A) The log_2_ fold-change values from whole genome re-sequencing data illustrating the read depth differences between the HB and HQLA breeds. This analysis validates the presence of the CNVs on GGA27 in chickens with the Muffs and beard phenotype that were previously identified using a CGH array experiment. (B) Schematic illustration of the CNV rearrangements in the *Mb* locus on GGA27. (C) Genes located within the three duplicated CNV regions include the 3’ sequence of *PSMC5* and entire *SMARCD2* in CNV1 (green shadow), entire *HOXB8* and *HOXB7* in CNV2 (blue shadow), 5’ sequence of *CCR7*, 3’ sequence of *KRT222*, and entire *SMARCE1* in CNV3 (pink shadow).

**Fig 4:**
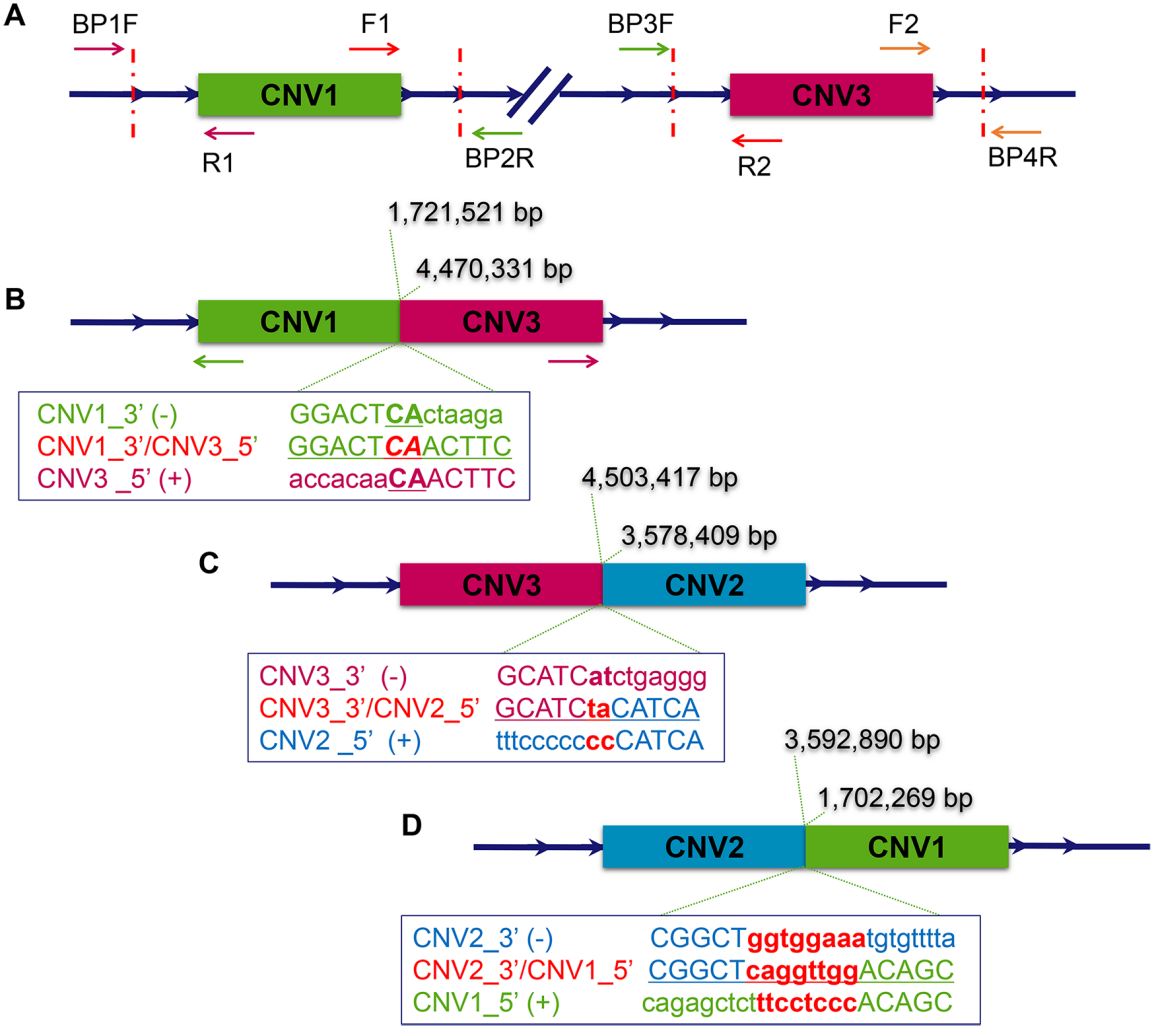
Analyzing the breakpoints of the copy number variations on GGA27 to clarify the rearrangement pattern. The duplicated regions identified by array-CGH are illustrated by green (CNV1), blue (CNV2) and pink (CNV3) boxes, respectively. (A) The boundaries of the CNVs were tested using 8 primers indicated by the arrows located in the known duplicated region of CNV1 and CNV3. All possible amplifications were considered and performed in both Mb and wild-type chickens. (B) The breakpoints of both CNV1_3’ (1,721,521 bp) and CNV3_5’ (4,470,331 bp) were identified after sequencing the specifically-amplified PCR product obtained in Mb chickens using primer F1 and R2. An overlap of two nucleotides was detected in the junction region. Outward facing primers (green and pink arrows) were designed to analyze the other boundary of CNV1 and CNV3. (C) CNV2 was found to be located next to CNV3 in chickens with the Mb phenotype using a genome-walking strategy. The breakpoints of both CNV3_3’ (4,503,417 bp) and CNV2_5’ (3,578,409 bp) were verified by sequencing. A two-nucleotide insertion was found in the junction region. (D) The breakpoints of both CNV2_3’ (3,592,890 bp) and CNV1_5’ (1,702,269 bp) were confirmed through unmapped read alignment of whole genome re-sequencing data. An eight-base insertion was detected in the junction.

In a previous study, we discovered two copy number variations (CNVs) in the genomic region harboring the *Mb* locus on GGA27 using array-based comparative genomic hybridization (array-CGH) with custom-designed 400k probes [19]. These polymorphisms, here denoted CNV1 and CNV3, were duplications of sequences located at 1.70 and 4.47 Mb on GGA27 that were present only in chickens with the Mb phenotype. The presence of these structural polymorphisms in, or close to, the *Mb* locus makes them interesting candidate variants for the Mb phenotype. An in-depth exploration of this region reveals i) that the *Mb* locus also contains a third CNV (CNV2) resulting from the duplication of a sequence located at 3.58 Mb of GGA27, ii) that CNV1 likely results from a tandem duplication, iii) that CNV2 and CNV3 are not tandem repeats, but rather duplications that are translocated between the CNV1 duplications (Fig 3). Together, these findings suggest this structural rearrangement as a strong candidate functional polymorphism in the *Mb* locus due to the overlap with the association and linkage peaks, as well as the IBD mapping result, in the region around 1.70 Mb on GGA27. Below, we described how these results were obtained and illustrated them in detail in Fig 4.

#### A copy of CNV3 is located near CNV1

To define the boundaries of the CNVs in the *Mb* locus on GGA27 and find the exact insertion loci, PCRs were performed in both *Mb/Mb* and *mb/mb* chickens with primers designed facing outwards from the CNV1 and CNV3 sequences that were known from our previous study [19] (Fig 4A). All possible combinations of these primers were considered, but only F1+R2 in the variant (*Mb*/*Mb*) chickens got a specific amplification, suggesting that a copy of CNV3 is located near CNV1 (Figs 3A and 4B). After sequencing the PCR products, we obtained the unilateral boundary of both CNV1 (CNV1_3’, 1,721,521 bp) and CNV3 (CNV3_5’, 4,470,331 bp) (Fig 4B). Amplifications with other primers in both *Mb/Mb* and *mb/mb* chickens showed that copies of both the CNV1 and CNV3 sequences were also located in their original sites on GGA27, as defined by the chicken reference genome.

#### A third CNV (CNV2) identified near one of the duplicated CNV3 sequences

Next, we adopted genome-walking using a combination of the primers described above and new primers designed for this purpose. After sequencing the specific products using primer F2, we identified a third copy number variation (CNV2), whose sequences originated from GGA27 3.58 Mb, next to CNV3. To further confirm this discovery, we re-analyzed the data from our previous array-CGH and discovered that CNV2 was also present there, but had been discarded due to the fact that only two unique probes covered this region (S2 Table). Sequencing of the PCR product identified the boundaries of CNV2 and CNV3 (CNV2_5’, 3,578,409 bp and CNV3_3’, 4,503,417 bp) (Figs 3A and 4C).

#### A duplicated CNV1 sequence is located next to CNV2

By aligning the unmapped reads from whole genome re-sequencing data from the HB and HQLA founder lines to GGA27, we could show that a copy of the CNV1 sequence was located next to CNV2 and define the boundaries of them (CNV2_3’, 3,592,890 bp and CNV1_5’, 1,702,269 bp) (Figs 3A and 4D). This discovery was confirmed by sequencing the specific PCR product amplified with primers designed according to the rearrangement. The re-sequencing data were also used to again verify the complex CNV1_CNV3 and CNV3_CNV2 junctions and the directions using the unmapped reads (S3 Table).

#### Re-confirming the order of duplications on the *Mb* allele at the *Mb* locus

To further confirm the presence of the three CNVs on GGA27, we generated whole-genome re-sequencing data based on the pooled samples from HB and HQLA populations separately. Read depth analysis showed a clear accumulation of reads at the three CNV loci (Fig 3A). By comprehensively evaluating all possible orders of the duplicated CNV sequences in the *Mb* locus, we confirmed the structure of this genomic variation. It involves duplication of three DNA segments joined together and inserted into the downstream of the original copy of CNV1 (Fig 3B).

### The structural variant on the *Mb* allele at the CNV1 locus is completely associated with the Mb trait

To test for association between the structural variant and the Mb phenotype, primers were designed to amplify sequences that were flanking and located within the region with the duplications at the *Mb* locus (S2A Fig). These were used to genotype 563 individuals from 31 chicken breeds and the HB × HQLA population (Table 1). Specific amplifications of this *Mb* allele were only detected in chickens with the Mb phenotype (S2B Fig), demonstrating a complete association between this SV and the Mb phenotype.

**Table 1:** Chicken used in PCR-based diagnostic tests of rearrangement.

Breed	Phenotype	
	Muffs and beard	Wild-type
**HH family**		
Huiyang Bearded (HB)	18	-
High Quality chicken Line A (HQLA)	-	18
HH^a^-F_1_	36	-
HH-F_2_	62	27
HH-F_5_	95	94
**Total**	211	139
**Muffs and beard**		
Beijing-You	11	-
Xiangdong	20	-
Yanzhou Bearded	6	-
Dutch Polish	2	-
Dutch Polish Bantam	1	-
Dutch Owl	12	-
Dutch Owl Bantam	9	-
Brabanter	7	-
Brabanter Bantam	11	-
**Total**	79	0
**Wild-type**		
White Leghorn	-	8
Beijing-You	-	12
Qingyuan Ma	-	6
White Ear	-	6
Wahui	-	6
Chahua	-	6
Henan Dou	-	6
Chongren Ma	-	6
Langshan	-	6
Bian	-	6
Luyuan	-	6
Tibet	-	6
Gushi	-	6
Dagu	-	6
Youxi Ma	-	6
Anka	-	6
White Recessive	-	6
Shouguang	-	6
Shiqiza	-	6
Aijiao Yellow	-	6
Red Jungle fowl	-	6
**Total**	0	134

^a^HH-F_n_: HB × HQLA intercross generation F_n_.

As all duplications are present in the forward direction, PCR reactions are unable to distinguish the *Mb/Mb* and *Mb/mb* genotypes. Therefore, we examined the duplicated region to locate copy-specific point mutations that would allow us to molecularly distinguish the two genotypes. This screen was performed using long-range PCR assays based on copy-specific amplification where each copy was divided into two parts and amplified in more than 23 individuals. Using comparative analysis of the mutations scored in this sequence data, as well as our re-sequencing data from nine Chinese non-Mb native chicken breeds, we identified a single SNP (a T-to-C substitution at 1,707,859 bp) that was copy-specific and can help distinguish *Mb/Mb* and *Mb/mb* genotypes in the Mb chickens (S3B Fig).

This T-to-C SNP was used as a genotyping marker through scoring the peak signal value of C and T at this locus by pyrosequencing. Only a T peak was detected in chickens homozygous for the recessive, wild-type allele (*mb/mb*), and equal peak values of T and C were detected in chickens homozygous for the dominant allele (*Mb/Mb*), while heterozygous chickens (*Mb/mb*) displayed a peak value of T that was twice that of C (S3C Fig). We genotyped 96 additional chickens (60 birds from our HB × HQLA population, 30 birds from 3 breeds displaying the Mb phenotype, and 6 non-Mb birds) for this polymorphism and found an excellent association between the Mb phenotype and this marker. These results provide additional evidence that the structural variant around 1.70 Mb that is specific to the *Mb* allele is actually the causal polymorphism at the *Mb* locus.

### Using expression-analysis to explore candidate genes at the *Mb* locus

The CNV regions that are duplicated in the Mb chickens contain a total of seven annotated genes. CNV1 contains partial proteasome 26S subunit, ATPase, 5 (*PSMC5*) and the entire SWI/SNF related, matrix associated, actin dependent regulator of chromatin, subfamily d, member 2 (*SMARCD2*), CNV2 contains the entire homeobox B7 (*HOXB7*) and homeobox B8 (*HOXB8*), and CNV3 contains partial chemokine (C-C motif) receptor 7 (*CCR7*), entire SWI/SNF related, matrix associated, actin dependent regulator of chromatin, subfamily e, member 1 (S*MARCE1*) and partial keratin 222 (*KRT222*) (Fig 3C). We next proceeded to evaluate these genes as functional candidates for the Mb phenotype by testing for their tissue-specific differential expression in the Mb and wild-type chickens.

#### Loss of *HOXB8* expression is detected in the facial skin of *mb/mb* chickens

Considering that the structural rearrangement on GGA27 might interfere with gene expression, we analyzed the transcripts of the seven genes located within the CNV regions using 5’RACE and 3’RACE with RNA extracted from the adult facial skin of *Mb/Mb* and *mb/mb* chickens.

**Fig 5:**
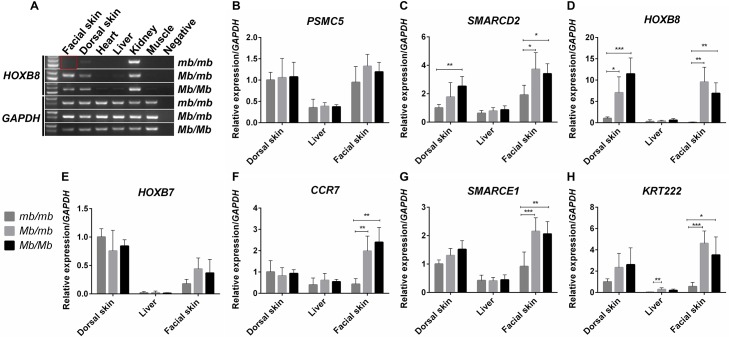
Differential expression of genes in the CNV regions in adult tissues. (A) The ectopic *HOXB8* expression in the facial skin was discovered by reverse-transcription PCR analysis. The negative control was non-template PCR reaction. The relative mRNA expression of (B) *PSMC5*, (C) *SMARCD2*, (D) *HOXB8*, (E) *HOXB7*, (F) *CCR7*, (G) *SMARCE1*, and (H) *KRT222* in the dorsal skin, liver and facial skin from adults were analyzed by qPCR. Barely detectable expression of *HOXB8* was also identified. All tissue samples used came from *Mb/Mb*, *mb/mb*, and *Mb/mb* adults. *Mb/Mb*, *mb/mb*, and *Mb/mb* represent *Mb* homozygous, wild-type homozygous, and heterozygous genotypes for the *Mb* locus regulating the Mb trait. ***p<0.001, **p<0.01, *p<0.05.

The transcript of *HOXB8*, a gene known to play an important role in feather development [20], was present in the facial skin tissue of Mb chicken, but not detectable in the facial skin tissue of wild-type chickens. We next used RT-PCR to verify this discovery in six tissues from chickens with different genotypes. In facial skin tissue, we observed ectopically high expression of *HOXB8* in Mb chickens (*Mb/Mb* & *Mb/mb*), but no expression in wild-type (*mb/mb*) chickens (Fig 5A). Validation of this finding in additional birds (S4 Fig) via RT-PCR strongly suggests a role for *HOXB8* in the morphogenesis of the Mb phenotype.

The transcripts of other six genes (*PSMC5*, *SMARCD2*, *HOXB7*, *CCR7*, *SMARCE1* and *KRT222*) were detectable in both wild-type and the Mb chickens, and no alternative splicing or variants between genotypes were found. We also confirmed through RT-PCR that all these six genes are expressed in facial skin tissue regardless the different genotypes of these chickens (S4 and S5 Figs).

#### An altered ectopic expression of *HOXB8* is a likely candidate mechanism leading to the Mb phenotype

To quantify possible differences in gene expression of the seven genes in the *Mb*-associated locus in embryonic, postnatal (two-week-old), and adult tissue, we measured their mRNA expression using quantitative RT-PCR (qRT-PCR). Expression was evaluated in both dorsal and facial skin.

**Fig 6:**
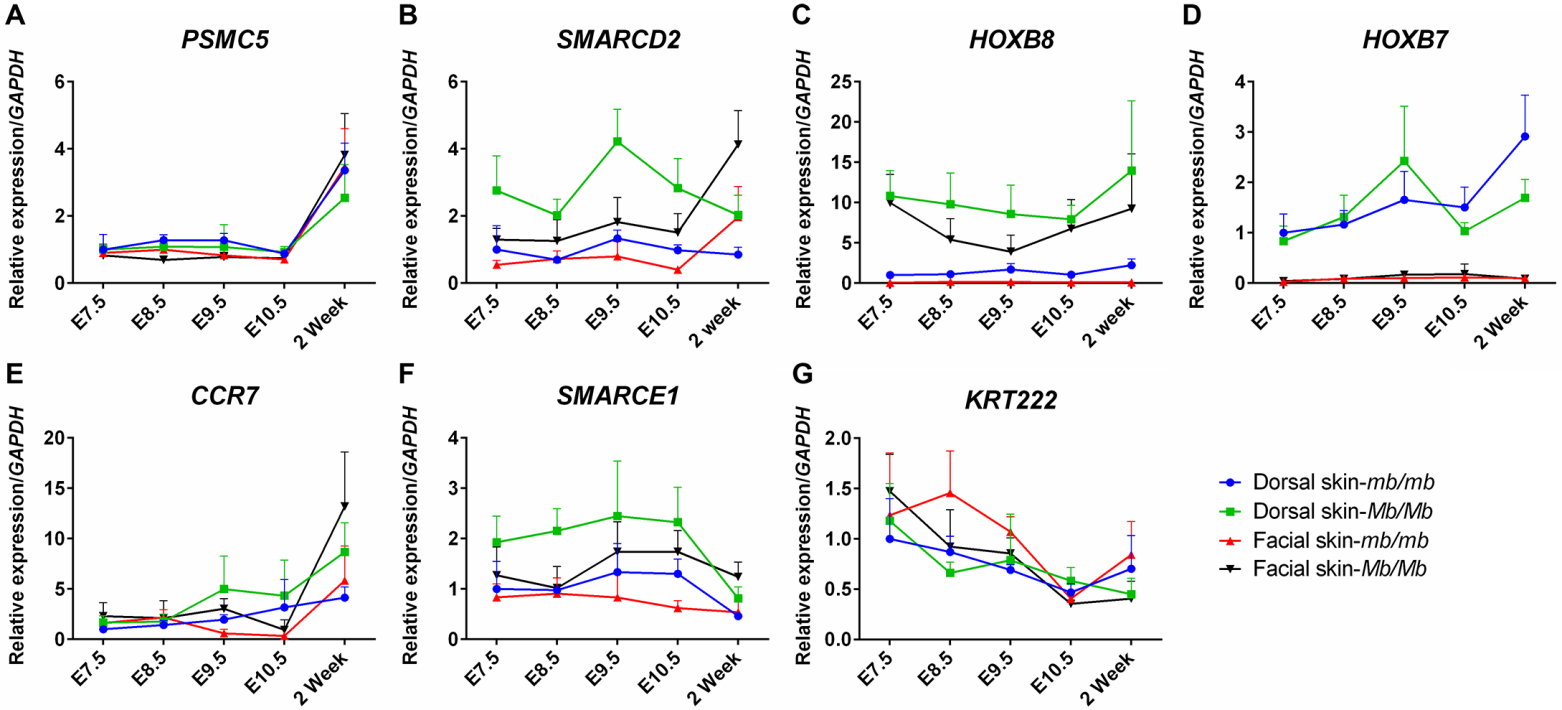
Relative gene expression during the critical stages of feather development. Gene expression analyses were performed in dorsal and facial skin tissue from embryos and chicks. Samples from *Mb/Mb* and *mb/mb* chickens were collected at embryonic (E) days 7.5, 8.5, 9.5, 10.5 and two weeks after hatching. The expression of (A) *PSMC5*, (B) *SMARCD2*, (C) *HOXB8*, (D) *HOXB7*, (E) *CCR7*, (F) *SMARCE1*, and (G) *KRT222* in the dorsal and facial skin were normalized to *GAPDH*. Ectopic expression of *HOXB8* was observed in the Mb chickens during the whole period of feather development.

The most pronounced differences that were coherent with the association to the Mb phenotype were observed for *HOXB8*. This gene showed a marked increase in expression in the facial skin of adult *Mb/Mb* and *Mb/mb* chickens, while being barely detectable in *mb/mb* chickens. Its expression was also increased considerably in the facial skin of developing *Mb*/*Mb* embryos and postnatal chicks. Differential expression of *HOXB8* was also observed in the dorsal skin during both developmental and adult stages, but unlike in facial skin, there was a basal expression of *HOXB8* in this tissue (Figs 5D and 6C).

Some degree of differential expression was also observed for the other genes in the region. The expression of *SMARCD2* increased in both dorsal and facial skin tissue of Mb chickens compared to wild-type controls. The altered expression was, however, not consistent with the Mb phenotype as the most pronounced difference was observed in the dorsal skin during development, and there was only a two-fold increase in expression in the facial skin of adult chickens (Figs 5C and 6B). Three more genes, *CCR7* (Figs 5F and 6E), *SMARCE1* (Figs 5G and 6F) and *KRT222* (Figs 5H and 6G), also displayed an increased expression in the facial skin of adult Mb chickens. However, this markedly increased expression was neither present in embryonic nor postnatal tissue. *PSMC5* did not show temporal and spatial differences in expression between genotypes (Figs 5B and 6A), and no statistically significant difference in expression in either the dorsal or facial skin was detected for *HOXB7* at any of the evaluated time-points (Figs 5E and 6D). In the livers of adults, the expression of all seven genes was low and only *KRT222* exhibited differences between *mb/mb* and *Mb/mb* chickens.

We also examined the expression of five other genes including *SCN4A*, *LOC771308*, *FTSJ3*, *ASIC2*, and *CD79B* in the 80 kb flanking region of CNV1. There were no significant differences between *mb* and *Mb* individuals in the facial skin (S6 Fig).

## Discussion

In the literature, the Mb has been described as a classic, autosomal, incompletely dominant trait [15,16]. This was also confirmed in our population since the relatively small Mb phenotypes were observed in the F_1_ and F_2_ *Mb/mb* chickens. Also, the intermediate phenotypes existed in Wattles of heterozygous chickens, and the distinct difference is easier to be seen in cocks than hens. Previous studies had observed that Wattles were absent or small when Mb was present [11,21]. Therefore, we speculate that the incompletely dominant inheritance of Mb trait is highly likely associated with the *Wattles* locus and there are underlying complex interactions for *Mb* and *Wattles*.

Recently, a gene mapping study in a cross between the Bejing-You chicken (BJY) and a commercial broiler line (Cobb-Vantress; CB) identified a single QTL for Mb on GGA27 [22]. Here, we used two populations from two different Chinese indigenous lines, Huiyang Bearded chickens (HB) and Beijing You chickens (BJY), both of which display the Mb trait, to dissect the genetic basis of this phenotype. As the Mb trait is fixed in the HB breed, whereas it still segregates in the BJY breed, we used two different experimental designs to exploit the two breeds for genotype-phenotype mapping. Using linkage (HB × HQLA F_2_ intercross) and association analysis (BJY population), we confirmed that only a single highly significant region on GGA27 contributes to the Mb trait. Using IBD mapping, we identified a target 48-kb region and further screened it for the causal mutation.

Genomic structural variation (SV) is an important part of genetic variations that could have a significant impact on gene expression and phenotypic variation. Many of such variations identified from gene mapping in domestic animals have illustrated the mechanisms behind expression changes and, as a result, influence the morphogenesis of a trait [23–26]. The regulatory patterns for SVs that influence phenotypic diversity include changes in i) gene dosage (more or less); ii) transcript (damage or rebuild); iii) regulatory element (gain or loss). With the advance of biochip technology, sequencing technology, and genome analysis tools, mutation detection at the genome level is now becoming experimentally routine, facilitating the high-resolution dissection of traits in many domestic animals [27–32]. But challenges in current studies still exist for identifying the complex SV itself in genomic regions and interpreting the relationship between it and its associated phenotypic variants.

Our study identified the *Mb* locus in chickens, which is an interesting example where a different type of structural variant contributes to the remarkable phenotypic diversity in domestic animals. Guided by the discovery of three duplication events, leading to CNVs that could be detected using array-hybridization in the *Mb* locus, we were able to identify a complex structural variation located around 1.70 Mb on GGA27. This SV was completely associated with the Mb phenotype in chickens and is highly likely the causal polymorphism on the *Mb* allele at this locus. It consisted of three duplications of segments located around 1.70 Mb (CNV1), 3.58 Mb (CNV2), and 4.47 Mb (CNV3) on GGA27, respectively. The duplications of the CNV2 and CNV3 sequences were translocated to the region between the tandemly located duplications of the CNV1 sequence, suggesting a highly complex event leading to this polymorphism. The order of the two copies of CNV1 was confirmed using data on a recombinant chromosome found in one of the F_2_-chickens. This bird had a wild-type phenotype (*mb/mb*), but a SNP genotype at GGA27 (1,707,859 bp) that was TC (T = C), rather than TT (S3A Fig). The recombination event was found to have happened near GGA27 (1,702,269 bp), which made it possible to infer which of the duplications was the original copy and which was the newly-acquired one (S3A Fig). Given that the association between the SNP at GGA27 (1,707,859 bp) and the Mb trait was not perfect, it is not the optimal candidate for genotyping. However, it is still a useful diagnostic marker for a first-step test to distinguish *Mb/mb* from *Mb/Mb*, as the false results can later be eliminated using a PCR-based diagnostic test and a phenotypic control, as wild-type chickens have neither the Mb trait nor the SV within their genome.

**Fig 7:**
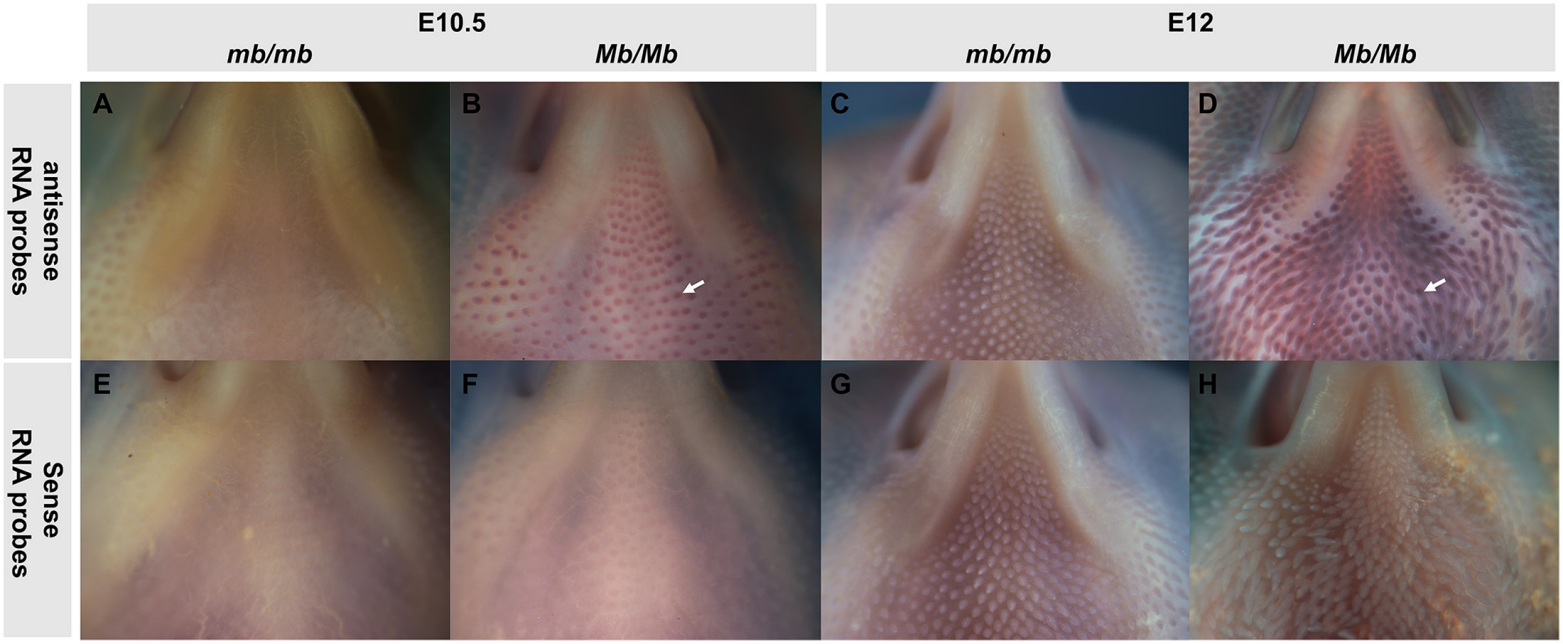
*In situ* hybridization scan of *HOXB8* expression in chicken embryos with different genotypes at the *Mb* locus. Skin samples in (A)—(D) were hybridized with *HOXB8* antisense probes, and in (E)—(H) were hybridized with sense probes as the negative control. The ectopic expression of *HOXB8* can be observed in the *Mb/Mb* chickens at the age of E10.5 and E12.

In total, seven genes were located in the duplicated segments in the *Mb* locus: *HOXB7*, *HOXB8*, *SMARCD2*, *SMARCE1*, *CCR7*, *KRT222*, and *PSMC5*. Although there were three genes (*PSMC5*, *KRT222*, and *CCR7*) truncated by the SV, it only carried the 3’ end of *PSMC5* and *KRT222* and the 5’ end of *CCR7* (Fig 3C). For *PSMC5* and *KRT222*: as shown in Fig 3C, the duplicated and translocated regions of CNV1 and CNV3 only contained the 3' sequences of *PSMC5* and *KRT222* genes (Fig 3C), which lack the promoter and regulatory sequences that required for gene transcription. Therefore, there seem no sufficient conditions for neither truncated *PSMC5* nor *KRT222* to transcribe and encode a new or a fusion transcript. As expected, we didn’t identify any unusual transcripts of both *PSMC5* and *KRT222* by the 5’ RACE using primers inside the SV region. For *CCR7*, according to the 3’RACE of *CCR7* using primers inside the SV region, we did not identify truncated mature mRNA in the *Mb/Mb* chickens. There are two possible reasons for the absence of detectable truncated mature mRNA of *CCR7*. First, the *CCR7* gene sequences inside the CNV3 region (the first 2 exons of *CCR7* gene, Fig 3C) do not contain necessary regulatory sequences for initiating its transcription in the examined skin tissue. Second, truncated mRNAs can be degraded by non-sense mediated decay pathway very rapidly in the cells. It is also possible that the truncated pre-mRNAs of *CCR7* are degraded rapidly in the cells. Using the qPCR primers inside the CNV region, we found that the significant difference of *CCR7* expression between *mb/mb* and *Mb/Mb* chickens only existed in 2-week-old and adult birds, but not in the stage of embryonic development. Since *Mb* birds are born with Muffs and beard, and the remarkable difference of the Mb trait was observed during embryonic development (Fig 7), genes that underlie the Mb trait probably show different expression levels between *mb/mb* and *Mb/Mb* chickens. Thus we infer that it is not *CCR7* that contributes to the Mb phenotype.

From a functional biology perspective, *HOX* genes are likely candidates to have a role in the development and differentiation of integumentary appendages [33]. Loss of *Hoxc13* expression in mice causes a fragile hair with an alopecia phenotype [34], and loss another *HOX* gene member, *Hoxb13*, promotes differentiation and enhances wound healing in adult skin [35]. *HOX* gene family encodes a family of transcription factors that have an important role in embryonic morphogenesis, such as establishing the anterior-posterior polarity of the early embryo [36,37]. *HOX* genes share strikingly conservative sequences and cluster together in the genome. The expression patterns of *HOX* genes in the vertebrate embryo are complex, usually present in a precise temporal and spatial collinear-manner, which is consistent with their positions in the *HOX* cluster [38–41]. The expression of some *HOX* genes in developing skin has been reported [42,43] and the function in the pattern formation and phenotypic determination of skin appendages in specific regions has been verified [39,44–47]. Based on these studies, *HOX* genes emerge as potential functional candidate regulators for feather/hair development and specifying regional identity of skin.

To investigate whether the structural *Mb* polymorphism might alter the expression of *HOXB7* or *HOXB8*, and in this way lead to the Mb phenotype, we performed gene expression analyses using qPCR tests in skin samples at successive stages of feather tract development. These were performed in embryos, 2-week-old chicks, and adults, and other genes present in the region were also included. The most pronounced differences in expression between Mb and wild-type chickens were found for *HOXB8*. It was completely repressed in the facial skin, and lowly expressed in the dorsal skin of wild-type chickens. Further, our *in situ* hybridization for *HOXB8* showed a similar result that apparent differences existed in Mb and wild-type chickens at the mRNA level (Fig 7). These results strongly suggested a role of this gene in Mb-feather development. Some differences at mRNA levels were also observed between Mb and wild-type chickens for the *HOXB7*, *SMARCD2*, *SMARCE1*, *CCR7*, *KRT222*, *PSMC5*, but the differences in expression levels were smaller for these genes and none displayed a Mb-specific expression pattern consistent with the establishment of the Mb phenotype during development.

The *HOX* genes generally have a regionally restricted expression in the skin and have been found in many cases to have a critical regulatory role in hair and feather development and patterning [44,48–50]. The regulatory mechanisms for these genes are complex and not yet fully understood, but studies on loss- or gain-of-function mutants in mice have provided some insight to the distribution of expression and mechanisms involved in the transcriptional regulation of the *HOX* genes [51–53]. The cluster organization of vertebrate *HOX* genes has also been found to have a critical regulatory role as it leads to *HOX* gene expression in a spatially co-linear manner [41,44,49], and mutations that disrupt the clustering or the regulatory module, have been found to cause transformations or defects in individual body patterning [53,54]. At the Mb locus, the duplication event of CNV2, leading to a duplication of *HOXB7* and *HOXB8*, did not include the conserved regulatory region inside the homeobox B cluster. Therefore, the expression of the *HOXB8* and *HOXB7* copies inside the duplicated CNV2 is no longer under the local control of the regulatory elements inside the original homeobox B cluster. The remarkably high ectopic expression of *HOXB8*, particularly in the facial skin of Mb chicken, might thus result from the loss of repression from the repressive elements inside the *HOX* cluster, potentially due to position specific effects induced by the duplicated CNV2 sequence. The expression of *HOXB7* was, however, not significantly altered, perhaps suggesting that the regulatory mechanisms are unique to each *HOX* gene to achieve the specificity in their temporal and spatial expression.

Previous studies that have explored the expression of *HOX* genes in the developing chicken [55], murine [46], and human skin [45] and have shown that the expression of *HOX* genes is body position specific. A hypothesis that the “*HOX* codes” influence site-specific epidermal differentiation through the spatial co-linearity manner and in this way contribute to the phenotypic determination of skin appendages [47] has been experimentally verified [56]. The conclusion from these studies is that members of the *HOX* family are involved in epithelial-mesenchymal tissue interactions during embryonic skin development and in this way influence the regional-specific skin appendages [57,58]. The role of *HOXB8* in development has been described in several earlier studies. It is an indispensable element in directing migration of the lateral line primordium [59] and expressed in various organs in human, mouse, and chicken. In mice, a loss-of-function mutation in *HOXB8* caused an obvious behavioral anomaly, leading to an excessive pathological grooming and hair removal at over-groomed sites [60]. Also, ectopic expression of *HOXB8* in the anterior margin of the forelimb bud has been found to cause a duplication of the zone of polarizing activity and a homeotic transformation of the axial structures [61]. At the molecular level, Ekert [62] has shown that down-regulation of *HOXB8* caused cell-cycle arrest and apoptosis in the presence of IL-3 in a majority of cells. This role of *HOXB8* in activating cell cycle and enhancing proliferation [62] supports our conclusion that over-expression of *HOXB8* was the cause of the Mb phenotype as feather development is a synergistic effect of cell proliferation and apoptosis. Feather development and regeneration is a conserved periodic process that involves growth (anagen), regression (catagen), and relative quiescence (telogen). The duration of each phase in follicles on different body sites give feather the variable lengths/sizes [63–66]. A similar variability in hair length is observed for the mouse hairy-ear phenotype, which is caused by a ~47 Mb inversion that doesn’t disrupt any protein-coding transcripts, but induces an elevated expression of the flanking *HOXC* gene in developing and mature skin of the ears [67]. In that study, it was suggested that the increased hair length might be due to an extension of the anagen stage in the hair cycle, thus implying a role of *HOXC* genes in hair-follicle cycle control. In the present study, the ectopic expression of *HOXB8* probably regulates the morphogenesis of the feather through lengthening the anagen phase of the follicles located on the chin. As a consequence, feathers are elongated and differentially developed. Since *HOXC8*, another *HOX* gene, whose ectopic expression is reported to have an association with *Crest* (a tuft of elongated feathers atop of the head) in chickens [3], we find it likely that the ectopic expression of specific posterior *HOX* genes in facial skin may also take part in the process of feather-lengthening and that there is likely some unknown mechanism by which the *HOX* genes serve as activators to regulate the feather growth cycles and influence morphology of feather during development.

In conclusion, our results demonstrated that the *Mb* allele leading to the Muffs and beard phenotype in chickens is the result of a complex structural variation on GGA27. Our results also strongly suggest that this allele leads to an altered ectopic expression of a homeotic gene *HOXB8*, to suggest a novel role for *HOXB8* in modulating the regional development of the feather. These results are another example of how striking phenotypes in domestic animals have facilitated the discovery of genomic structural variants that alter developmental phenotypes. In addition to an increased understanding about the genetic mechanisms contributing to feather-development and differentiation, it also presents an excellent model for exploring the basic mechanisms underlying the spatial and temporal regulation of *HOX* genes.

## Materials and Methods

### Ethics statements

This study was approved by the Animal Welfare Committee of China Agricultural University. The approval number is SKLAB-2013-0605. All chickens used in this study were taken care and operated according to the relevant regulations.

### Animal material

Animals used for the association mapping came from two populations: one is the HB (Huiyang Bearded chicken, an Mb broiler line) × HQLA (High Quality of Chicken Line A, a non-Mb broiler line) population including 511 F_2_, 52 F_1_, and 22 F_0_ [17], and the other is the Beijing-You population [18] (a typical Chinese local breed) including 724 individuals. The birds used for linkage analysis and IBD-mapping as well as subsequent gene expression analyses were from the HB × HQLA population. After the second (F_2_) generation, a deep intercross HB × HQLA population was bred by random mating. F_7_ chickens were mated to produce the F_8_ embryos and chicks for the gene expression studies.

### Mapping of the *Mb* locus

Genotyping was performed using an Illumina chicken 60k SNP Beadchip. Quality control was firstly performed using the following criteria: call rate of individuals >0.9 and call frequency of SNPs >0.9. Markers with a minor allele frequency >0.05 were also removed. Then, as we had full pedigree records of the HB × HQLA cross, we identified putative genotyping errors that did not follow Mendelian inheritance. Entire birds or SNPs were excluded from the data if they had a higher than 5% error rate in the pedigree-based inheritance examination, and otherwise, only the individual data points were removed. Finally, we checked the SNPs on the sex chromosomes, and all 7 SNPs on chromosome W were removed due to their low call rate. For SNPs on chromosome Z, we checked that no heterozygous genotypes were present in hens, indicating that the genotypes are consistent with hens being the heterogametic sex (ZW). In total, 24 individuals and 15,239 SNPs were excluded as they did not fulfil at least one of the above criteria. The Mb phenotype was recorded every two weeks after hatching until 12 weeks of age. Individuals that had missing values in their records were removed from the dataset. Also, as the Mb feathers grew during this time, any individual’s record showing inconsistency with such a trend was removed. In total, 564 birds and 43,493 SNPs were included in the later mapping studies. A linear mixed model was used for the genome-wide association analysis of the Mb trait at 10 weeks of age and was performed using the GenABEL package in R [68–71]. The Mb trait at birth was included as a covariate in the analysis, and the genomic kinship matrix was used to account for familiar relatedness. To test for a possible second independent associated locus on GGA27, we performed a second association analysis where the genotype of the most significant SNP was included as a covariate in the model. Since no second significant peak was found, we concluded that the association was likely to be due to a single-locus. Bonferroni correction was used to set the genome-wide significance threshold.

For linkage and IBD analyses, we first constructed a genetic map for the HB × HQLA intercross population using an improved version of the CRI-MAP software [72], and then computed the line-origin probabilities at each centiMorgan (cM) using the trim algorithm [73]. The linkage analysis was performed using MAPfastR [74] with the Mb trait at birth as a covariate. The genome-wide significance threshold was determined using a 1000-fold randomization test. Based on the line-origin probabilities, we deduced the line-origin of chromosomal segments across GGA27 for each F_2_ chicken. Using a shared IBD analysis, the *Mb* locus was fine-mapped by finding the shortest overlap between the chromosomal segments that originated from the HB line and that were shared between all F_2_ chickens with the Mb phenotype, but that did not occur in non-Mb chickens.

### PCR-based screens for the genomic rearrangement

Outward facing primers and genome-walking were used to analyze the breakpoints and insertion loci for the CNVs in the *Mb* locus. PCR primers for diagnostic tests were designed according to the uncovered genomic rearrangement (Figs 3 and 4) located on either side of the evidenced breakpoints. All the samples (Table 1) used in diagnostic tests were collected in China except Dutch Polish, Dutch Polish Bantam, Dutch Owl, Dutch Owl Bantam, Brabanter and Brabanter Bantam which were collected in the Netherlands. DNA was extracted from blood using DNeasy Blood & Tissue Kit (Qiagen) and diluted to 50 ng/μl. PCR products were examined using 2% agarose gel electrophoresis.

### Whole-genome re-sequencing

DNA from pools of individuals from the founder lines for the F_2_ intercross was whole-genome re-sequenced. For the pools, HB (n = 15) and HQLA chickens (n = 16) were used. Sequencing libraries (170-bp paired-end, 400-bp paired-end and 3-kb mate-pair) were constructed, and whole-genome re-sequencing was performed using the Illumina HiSeq 2000 platform by BGI (Shenzhen). Sequence reads were mapped against the ICGSC Gallus_gallus-4.0 reference genome (Nov. 2011) using BWA [75]. The average read depth was estimated using GATK [76] (version 3.1–1) to 95 × for the HB chicken sample and 97 × for the HQLA sample. Read depth differences between two breeds were calculated using a 1 kb sliding window strategy. To reduce false positive results, low mapping quality regions (1-kb window average MQ < 30, the threshold value was set to the mean MQ value of 1-kb genome bin minus 3 standard deviations) were filtered. The whole-genome sequencing data had been deposited in the SRA database at NCBI with a BioProject accession number PRJNA306810.

### Re-sequencing of targeted genomic regions

Re-sequencing of the three identified CNVs sequences was performed using long-range PCR and Ion Torrent Technology. The samples used included 4 F_2_-birds from the HQ × HQLA population (*Mb/Mb*, n = 1; *mb/mb*, n = 3), 6 homozygotes from 3 Mb breeds (Xiangdong, Beijing-You, Huiyang Bearded chicken), and 13 birds from 13 wild-type breeds (Red jungle fowl, Wenchang, Qingyuan ma, White ear, Wahui, Chahua, Henan Dou, Chongren ma, Langshan, Bian, Tibet, Anka, and Recessive white). Each CNV-region and the two copies of these were divided into two parts and were amplified using the primers listed in S5 Table. LongAmp Taq DNA Polymerase (New England Biolabs) was used in the long-range PCR amplification. Amplified PCR fragments were purified using the Gel Extraction kit (OMEGA Bio-Tek) and equimolarly mixed. Then, libraries were constructed, and the samples were sequenced using Ion torrent 314 and 316 chips using standardized protocols for Ion torrent sequencing. BWA was used for alignment [75], and samtools was used for mutations detection [77].

### Genotyping using pyrosequencing

Genotyping of the T-to-C substitution at 1,707,859 bp that differentiated the *Mb* and *mb* alleles based on the presence of the duplication of CNV1 or not was performed using pyrosequencing with primers pyro-forward (5'-TCTGCCCCTGTTCTGTACCAT-3'), pyro-biot-reverse (5'-Biot-AGCTGCGTGGGCTGAAAC-3') and pyro-seq (5'-ACCCAACAGCCTCCC-3'). A 92-bp-DNA-segment was amplified with pyro-forward and pyro-biot-reverse primers to prepare the biotinylated single-stranded PCR amplicon for pyrosequencing. Genotypes were determined by the peak value of the signals and calculated using Pearson's chi-squared test (P = 0.05). We used this method to examine F_1_ (n = 12), F_2_ individuals including *Mb/Mb* (n = 24), *Mb/mb* (n = 12), *mb/mb* (n = 12) chickens in the HB × HQLA cross, and birds from other breeds including Xiangdong (*Mb/Mb*, n = 12), Beijing-You (*Mb/Mb*, n = 6; *mb/mb*, n = 6), Huiyang Bearded (*Mb/Mb*, n = 6), and Shouguang (*mb/mb*, n = 6) to confirm our discovery. Further, we also genotyped a total of 112 F_7_ chickens for the follow-up gene expression experiments.

### Reverse transcription PCR (RT-PCR)

We examined six different adult tissues (facial skin, dorsal skin, heart, liver, kidney, and muscle) collected from F_8_-individuals (*Mb/Mb*, n = 1; *Mb/mb*, n = 1; *mb/mb*, n = 1) for the gene expression analysis. The skin samples used in the expression analysis were dissected from the two anatomical sites that were listed in S7 Fig. The verification of the expression of *HOXB8* in the facial skin was performed in additional F_8_-individuals (*Mb/Mb*, n = 7; *Mb/mb*, n = 7; *mb/mb*, n = 7). RNA was extracted using RNeasy Mini kit (Qiagen). cDNA was synthesized using M-MLV Reverse Transcriptase and RNasin Ribonuclease Inhibitor (Promega) with 1 μg total RNA in a 20 μl volume. RT-PCR was performed in a total 25 μl volume using 1 μl of reverse transcription products as templates.

### RACE

RNA samples from the adult facial skin of the HB × HQLA family generation F_7_ were used in RACE. 5’ RACE was performed using the 5’ RACE System for Rapid Amplification of cDNA Ends, Version 2.0 (Invitrogen). A gene-specific primer was used to synthesize the first strand cDNA. After purification of the first strand product, a homopolymeric tail was added to the 3' ends of the cDNA. With two gene-specific primers, nested PCR reactions were performed to amplify the 5’ end of the target gene. For 3’ RACE, cDNAs were synthesized with 2 ug mRNA using reverse transcriptase and an oligo-dT adapter primer. Gene-specific cDNAs were then directly amplified using two gene specific primers and two universal amplification primers. The PCR products were cloned into T-vector and sequenced using the Sanger method.

### Real-time qRT-PCR

Skin samples were collected from generation F_8_ of the HB × HQLA intercross including embryos at embryonic day (E) 7.5, 8.5, 9.5 and 10.5, chicks of two-week-old and adult chickens. RNAs derived from embryo tissue were isolated using RNeasy micro kit (Qiagen), while RNAs from other tissue were homogenized in TRIzol (Invitrogen) followed by DNase I treatment and clean-up using the RNeasy mini kit (Qiagen). cDNAs were generated with 1 μg RNA using Reverse Transcription System (Promega) in a 20 μl volume. The cDNAs were then applied to specific target amplifications, respectively, using a forward and reverse primer mix with each primer at a concentration of 100 μM in a 5 μl volume to increase the number of copies of target genes. Then a cleanup step with Exonuclease I was performed to remove unincorporated primers, and the final products were diluted before qPCR reactions. After loading samples and assays into the Dynamic Array IFC (Fluidigm), cDNA levels were quantified by qPCR using SsoFast EvaGreen Supermix (Bio-Rad) with Fluidigm Biomark HD system. Samples were run in sextuplicate using EvaGreen Supermix (Bio-rad) and normalized to *GAPDH*. The 2^-ΔΔCT^ method was used to analyze the relative changes in gene expression. Statistical analysis was performed with GraphPad Prism 6 (GraphPad Software, San Diego, CA).

### Whole mount *in situ* hybridization

Whole-mount *in situ* hybridization (ISH) was performed following the standard procedures of GEISHA (http://geisha.arizona.edu/geisha/) using the *HOXB8* probe (571 bp) listed on the website. Embryos were collected at E10.5 and E12 at room temperature and washed in calcium-magnesium free PBS. After overnight fixation in 4% paraformaldehyde/PBS-2mM EGTA at 4°C, the embryos were dehydrated with a graded methanol series. The hybridization was done at 70°C for 48 hours with a probe concentration of 200 ng/ml. Embryos were washed to remove unbound probes and blocked in 20% heat inactivated (55°C for 30 min) sheep serum solution for 3 h at room temperature. After incubating embryos in anti-digoxigenin antibody conjugated to alkaline phosphatase (Roche) overnight at 4°C, hybridization was detected using a BCIP/NBT color reaction (AMRESCO).

## Supporting Information

S1 Fig Manhattan plot of genome-wide association analysis for the Mb phenotype in Beijing-You chickens. (A) Manhattan plot for the Genome-wide association analysis of the Mb trait in Beijing-You chickens. The x-axis shows the chromosome position, and the y-axis shows the -log_10_ *p* values. (B) A scatter plot for all SNPs tested on GGA27. The peak SNP (rs14301648: GGA27 at 1,745,051 pb) is marked with a filled triangle.
(TIF)

S2 Fig PCR-based diagnostic tests of the structural rearrangement. (A) The structural rearrangement on GGA27 was detected using three pairs of primers. Primer CNV1_3_F & CNV1_3_R, CNV3_2_F & CNV3_2_R, and CNV2_1_F & CNV2_1_R were used to amplify a 3138-bp, a 501-bp, and a 411-bp fragment respectively. (B) Gel images of electrophoresed PCR products from Mb (n = 6) and mb (n = 6) F_2_ individuals. Amplification was detected in all the Mb chickens. No amplification was detected in the wild-type chickens.
(TIF)

S3 Fig Long-range PCR based mutation analysis and pyrosequencing based genotyping. (A) The special F_2_ (96083) bird is a non-Mb chicken from HB × HQLA population. The recombination event occurred during the gametogenesis of its mother. And it resulted in an allele containing part of paternal Mb chromosome started from the recombinant site (1,702,798 bp). Therefore, its genotype at GGA27:1,707,859 bp was T/C instead of T/T. (B) The CNV regions were divided into two parts, and copy-specific mutations were analyzed by long-range PCR. (C) The genotyping results of the copy-specific SNP (1,707,859 bp) were performed using pyrosequencing.
(TIF)

S4 Fig Semi-quantitative reverse-transcription PCR analysis of the expression of all genes in the CNV regions in more individuals. (TIF)

S5 Fig Semi-quantitative reverse-transcription PCR analysis of the expression of all genes except *HOXB8* inside the CNV regions. Semi-quantitative reverse-transcription PCR analyses of gene (*HOXB7*, *CCR7*, *KRT222*, *PSMC5*, *SMARCD2*, and *SMARCE1*) expression levels in the facial and dorsal skin, heart, liver, kidney and muscle were detected in *mb/mb*, *Mb/mb*, and *Mb/Mb* chickens respectively.
(TIF)

S6 Fig Expression of the genes in the region flanking CNV1. The relative mRNA level of (A) *CD79B*, (B) *SCN4A*, (C) *LOC771308*, (D) *FTSJ3* and (E) *ASIC2* in the dorsal skin, facial skin, liver, kidney, heart, and muscle.
(TIF)

S7 Fig Anatomical sites of skin samples used in the gene expression analysis. The facial skin used in the gene expression analyses was dissected from the triangular region, illustrated on the left, whereas the dorsal skin was dissected from the rectangular region shown on the right.
(TIF)

S1 Table Genome-wide association results for the Mb trait in the two analyzed populations. (DOCX)

S2 Table CNVs identified by array-CGH. (DOCX)

S3 Table Alignment of unmapped reads to validate the rearrangement. (DOCX)

S4 Table Product length of long-range PCRs. (DOCX)

S5 Table Primer information. (DOCX)
