# Supplementary figures and images for: A Complex Structural Variation on Chromosome 27 Leads to the Ectopic Expression of HOXB8 and the Muffs and Beard Phenotype in Chickens
Source: PLoS Genet. 2016 Jun 2;12(6):e1006071. doi: 10.1371/journal.pgen.1006071 (PMC4890787; doi:10.1371/journal.pgen.1006071)

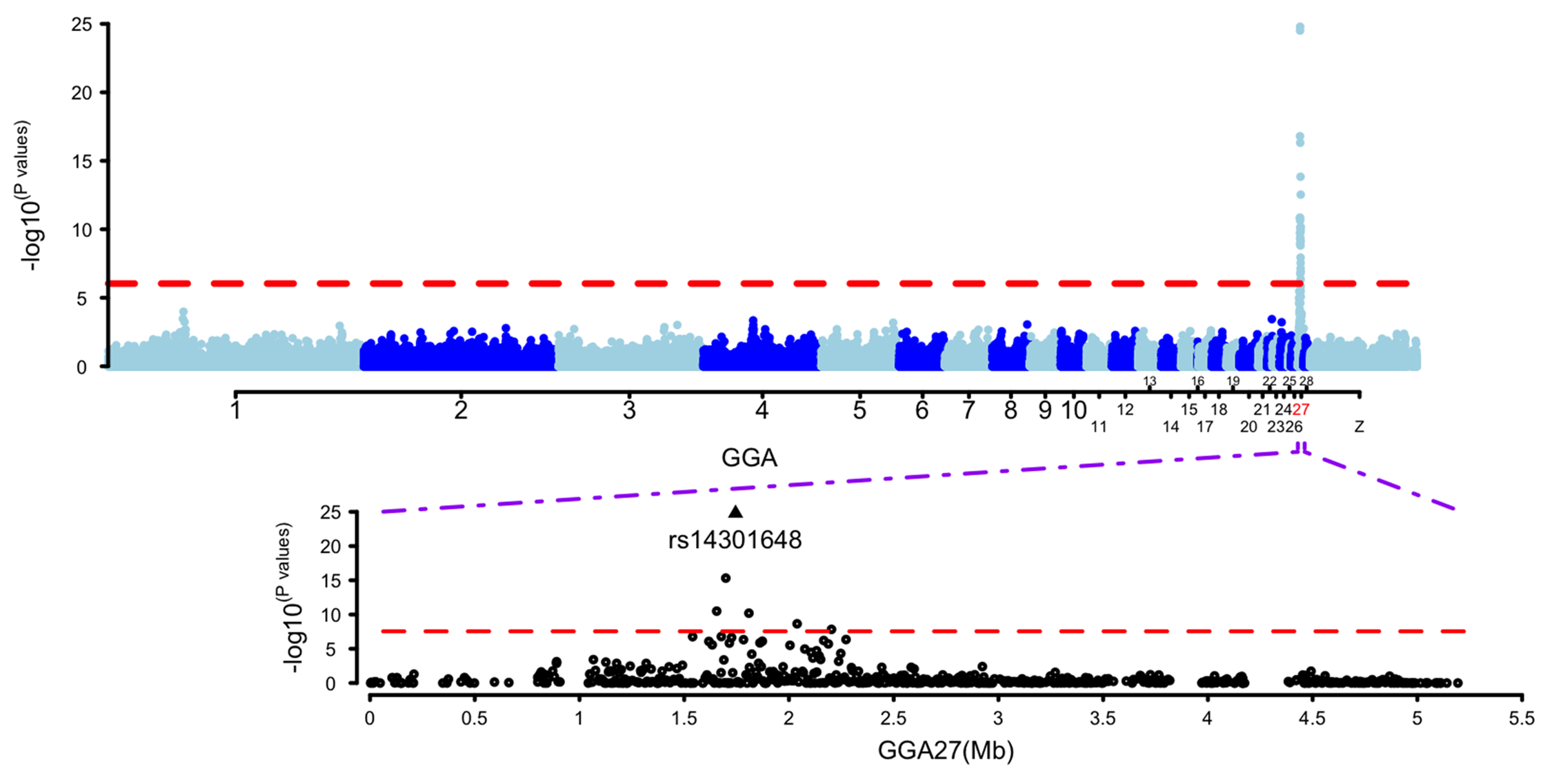

Supplement: S1 Fig — (A) Manhattan plot for the Genome-wide association analysis of the Mb trait in Beijing-You chickens. The x-axis shows the chromosome position, and the y-axis shows the -log10 p values. (B) A scatter plot for all SNPs tested on GGA27. The peak SNP (rs14301648: GGA27 at 1,745,051 pb) is marked with a filled triangle. (TIF) [file pgen.1006071.s001.tif]

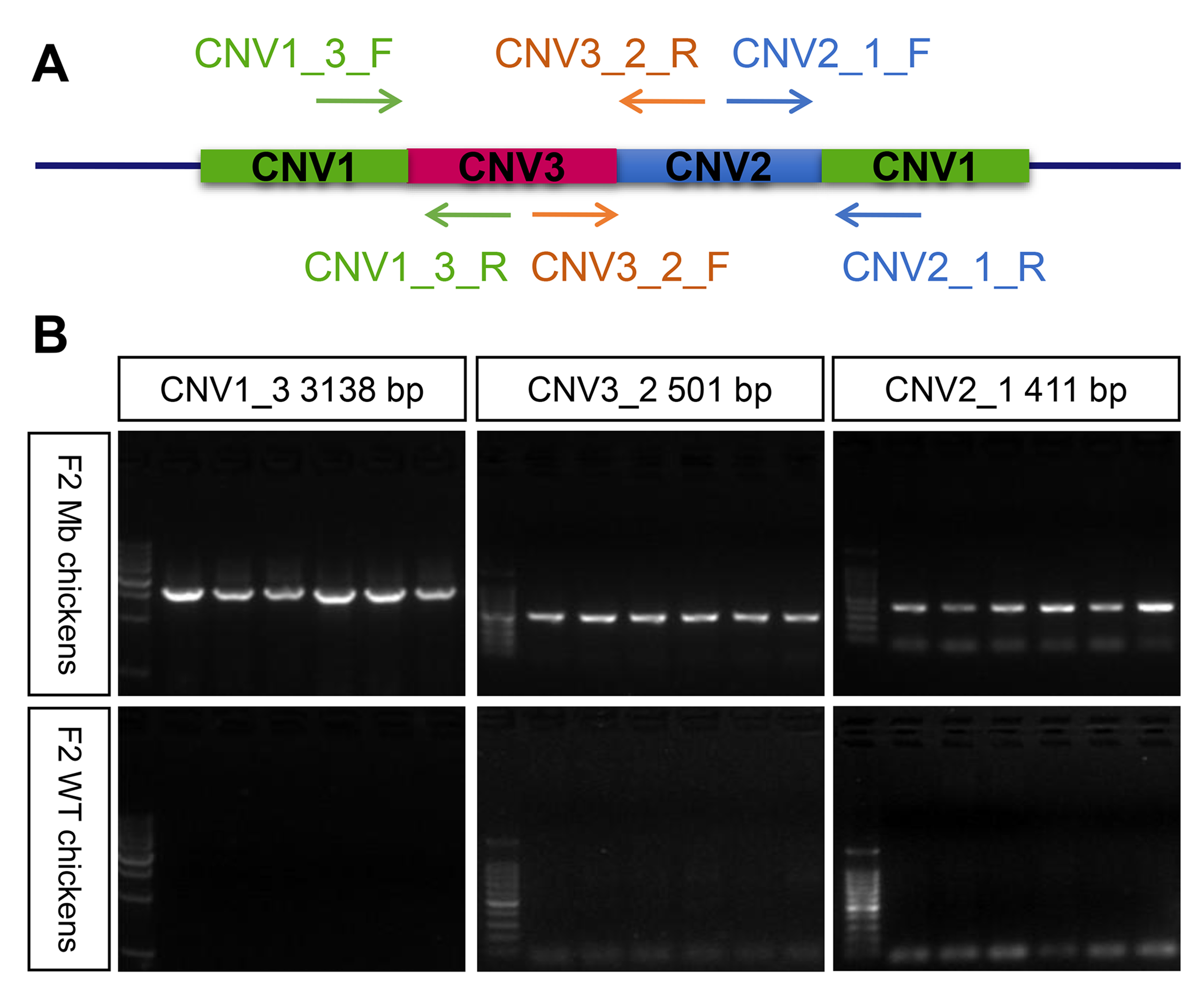

Supplement: S2 Fig — (A) The structural rearrangement on GGA27 was detected using three pairs of primers. Primer CNV1_3_F & CNV1_3_R, CNV3_2_F & CNV3_2_R, and CNV2_1_F & CNV2_1_R were used to amplify a 3138-bp, a 501-bp, and a 411-bp fragment respectively. (B) Gel images of electrophoresed PCR products from Mb (n = 6) and mb (n = 6) F2 individuals. Amplification was detected in all the Mb chickens. No amplification was detected in the wild-type chickens. (TIF) [file pgen.1006071.s002.tif]

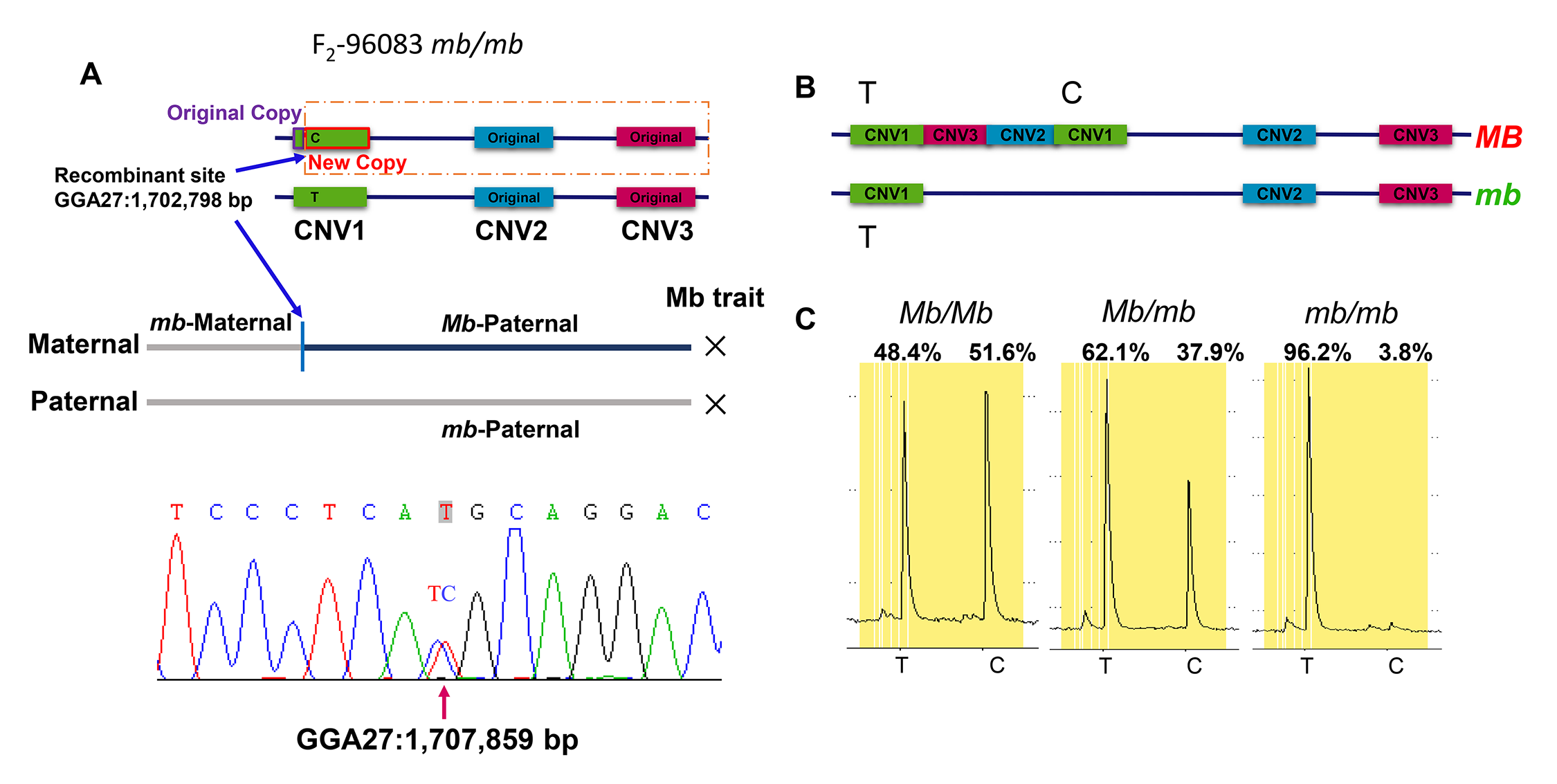

Supplement: S3 Fig — (A) The special F2 (96083) bird is a non-Mb chicken from HB × HQLA population. The recombination event occurred during the gametogenesis of its mother. And it resulted in an allele containing part of paternal Mb chromosome started from the recombinant site (1,702,798 bp). Therefore, its genotype at GGA27:1,707,859 bp was T/C instead of T/T. (B) The CNV regions were divided into two parts, and copy-specific mutations were analyzed by long-range PCR. (C) The genotyping results of the copy-specific SNP (1,707,859 bp) were performed using pyrosequencing. (TIF) [file pgen.1006071.s003.tif]

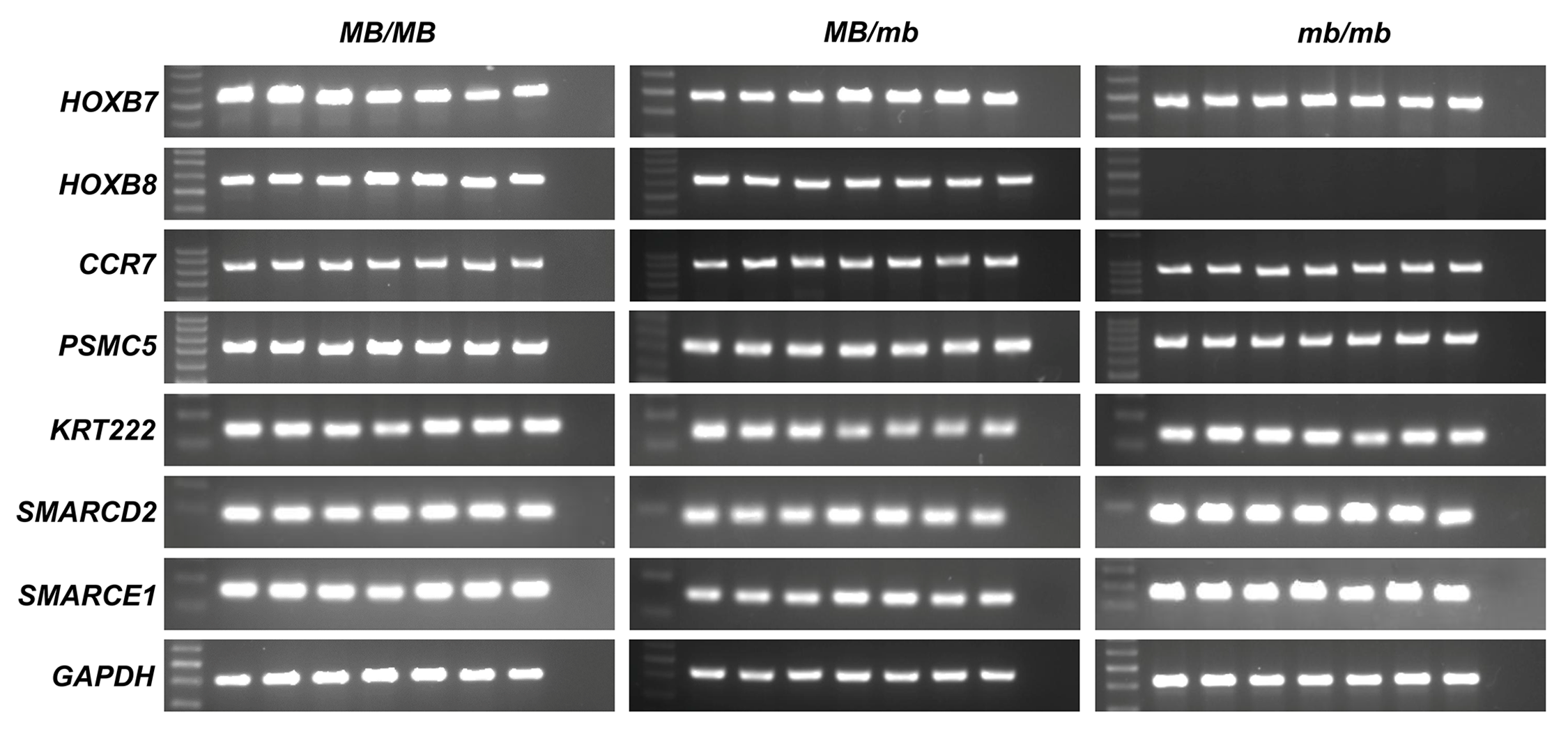

Supplement: S4 Fig — (TIF) [file pgen.1006071.s004.tif]

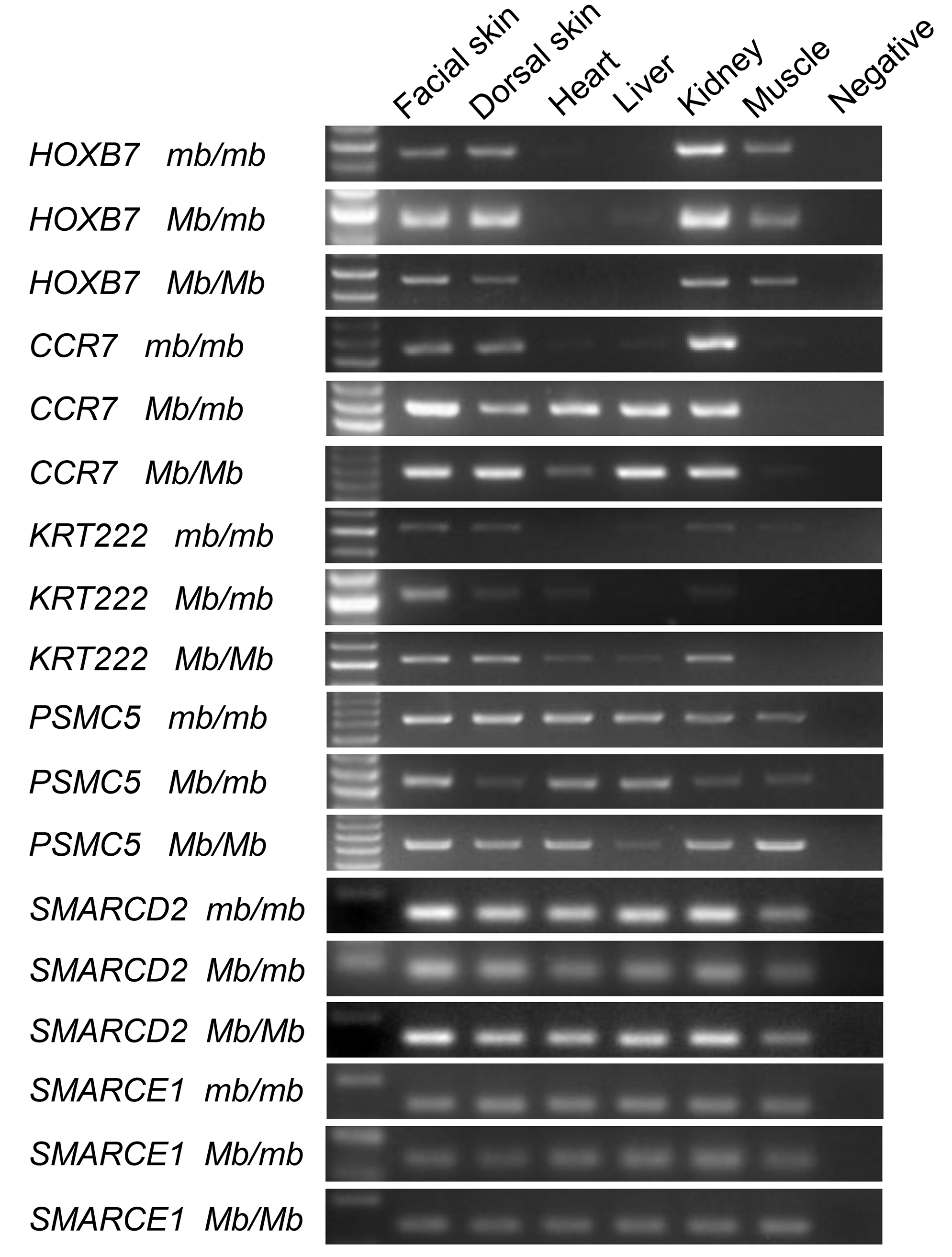

Supplement: S5 Fig — Semi-quantitative reverse-transcription PCR analyses of gene (HOXB7, CCR7, KRT222, PSMC5, SMARCD2, and SMARCE1) expression levels in the facial and dorsal skin, heart, liver, kidney and muscle were detected in mb/mb, Mb/mb, and Mb/Mb chickens respectively. (TIF) [file pgen.1006071.s005.tif]

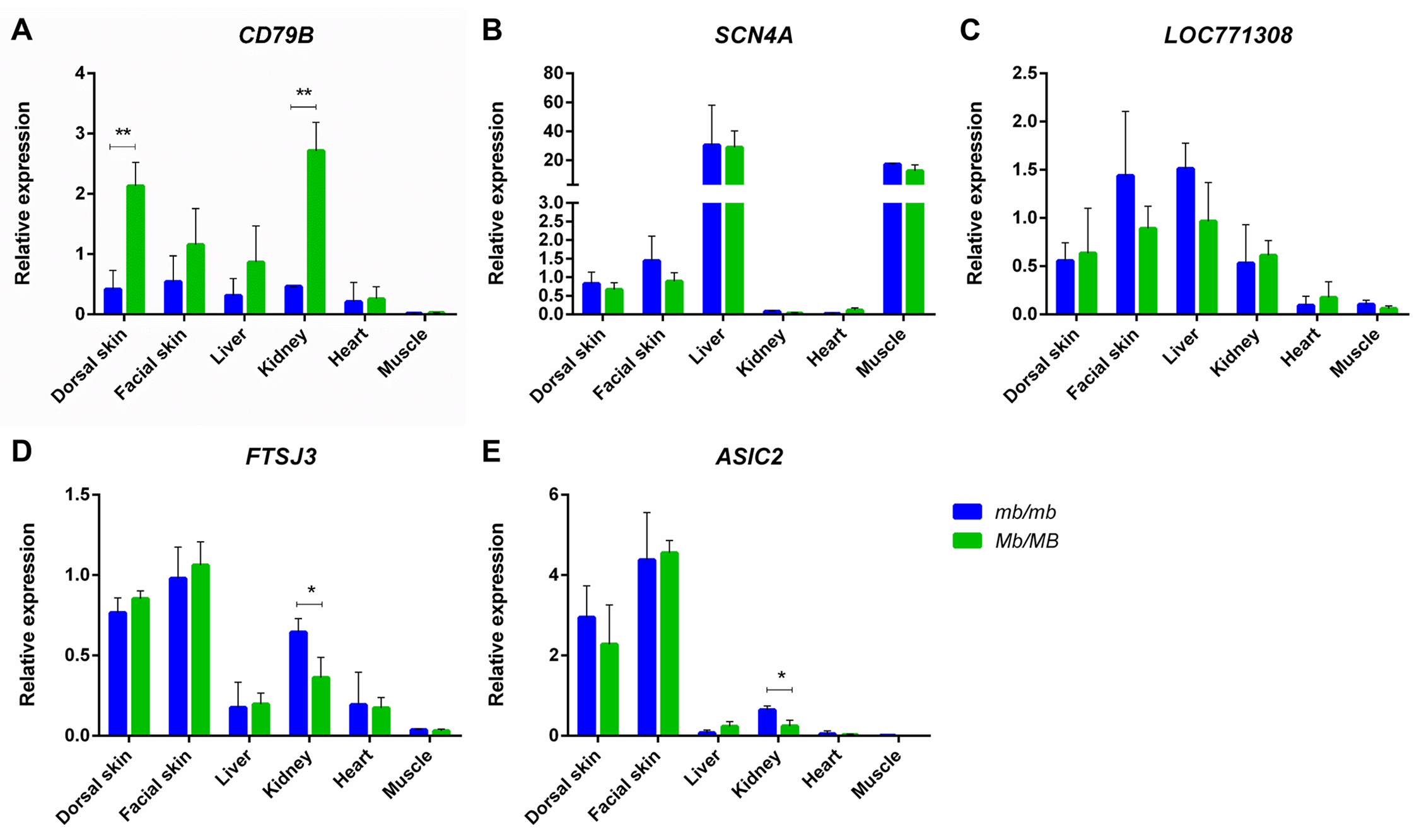

Supplement: S6 Fig — The relative mRNA level of (A) CD79B, (B) SCN4A, (C) LOC771308, (D) FTSJ3 and (E) ASIC2 in the dorsal skin, facial skin, liver, kidney, heart, and muscle. (TIF) [file pgen.1006071.s006.tif]

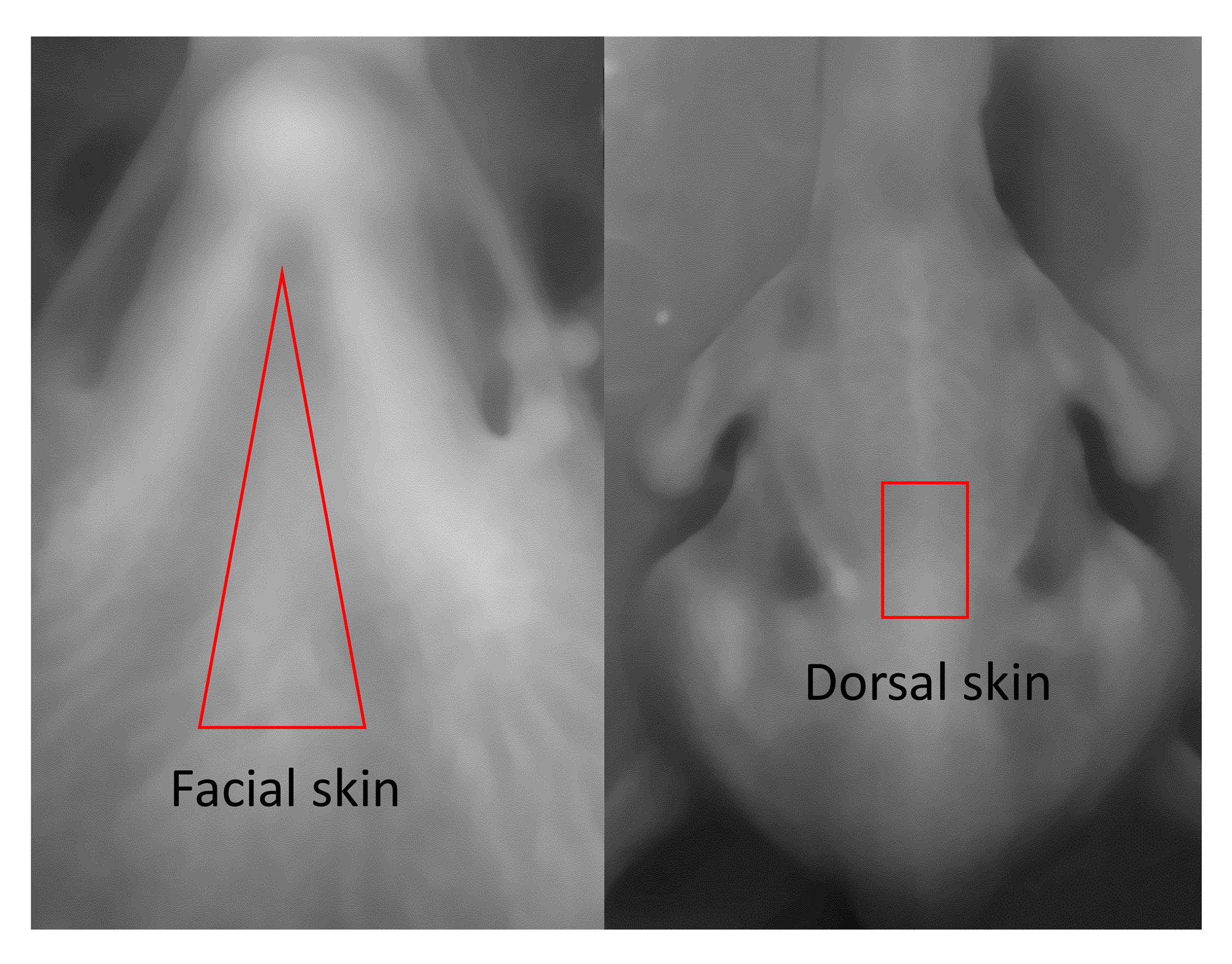

Supplement: S7 Fig — The facial skin used in the gene expression analyses was dissected from the triangular region, illustrated on the left, whereas the dorsal skin was dissected from the rectangular region shown on the right. (TIF) [file pgen.1006071.s007.tif]
