# Supplementary material for: A Complex Structural Variation on Chromosome 27 Leads to the Ectopic Expression of HOXB8 and the Muffs and Beard Phenotype in Chickens
Source: PLoS Genet. 2016 Jun 2;12(6):e1006071. doi: 10.1371/journal.pgen.1006071 (PMC4890787; doi:10.1371/journal.pgen.1006071)
Supplement: S1 Table — (DOCX) [file pgen.1006071.s008.docx]

**Table S1.** Genome-wide association results for the Mb trait in the two analyzed populations

| Population | Genotype source | Threshold | Peak SNP  (p value) | Significant region (Mb) |
| --- | --- | --- | --- | --- |
| HB × HQLA^a^ | 60K SNP chip | 5% genome-wide Bonferroni correction | rs13620154 (7.4×10^-20^) | 1.1-3.5 |
| Beijing Fatty chickens | 60K SNP chip | 5% genome-wide Bonferroni correction | rs14301648 (1.62×10^-25^) | 1.5-2.1 |

^a^: indicates the resource population built by crossing the Huiyang Bearded chicken (HB) and a non-Mb broiler line (High Quality chicken Line A or HQLA)
