# Supplementary material for: A Complex Structural Variation on Chromosome 27 Leads to the Ectopic Expression of HOXB8 and the Muffs and Beard Phenotype in Chickens
Source: PLoS Genet. 2016 Jun 2;12(6):e1006071. doi: 10.1371/journal.pgen.1006071 (PMC4890787; doi:10.1371/journal.pgen.1006071)
Supplement: S2 Table — (DOCX) [file pgen.1006071.s009.docx]

**Table S2.** CNVs identified by array-CGH^a^

| **ID** | **Chromosome** | **Start (bp)** | **Stop (bp)** | **Size (bp)** | **Unique Probes** | **Gains** |
| --- | --- | --- | --- | --- | --- | --- |
| CNV-1 | chr27 | 1,701,081 | 1,720,386 | 19,305 | 11 | 2 |
| CNV-2 | chr27 | 3,581,044 | 3,583,446 | 2,402 | 2 | 2 |
| CNV-3 | chr27 | 4,471,823 | 4,503,373 | 31,550 | 17 | 2 |

^a^: the raw CGH data has been deposited into the GenBank GEO database (GSE36504)
