# Supplementary material for: A Complex Structural Variation on Chromosome 27 Leads to the Ectopic Expression of HOXB8 and the Muffs and Beard Phenotype in Chickens
Source: PLoS Genet. 2016 Jun 2;12(6):e1006071. doi: 10.1371/journal.pgen.1006071 (PMC4890787; doi:10.1371/journal.pgen.1006071)
Supplement: S3 Table — (DOCX) [file pgen.1006071.s010.docx]

**Table S3.** Alignment of unmapped reads to validate the rearrangement.

| **Index** | **Read length** | **Start** | **End** | **Chromosome** | **Identity** | **Mismatch** | **Start** | **End** |
| --- | --- | --- | --- | --- | --- | --- | --- | --- |
| CNV1_CNV3 |  |  |  |  |  |  |  |  |
| FCD115HACXX:6:1202:15492:171184#TCTTATAT | 33 | 1 | 33 | chr27 | 100 | 0 | 4,470,363 | **4,470,331** |
| FCD115HACXX:6:1202:15492:171184#TCTTATAT | 69 | 32 | 100 | chr27 | 100 | 0 | **1,721,521** | 1,721,453 |
| FCD115HACXX:6:1208:14444:29719#TCTTATAT | 60 | 1 | 60 | chr27 | 100 | 0 | 4,470,390 | **4,470,331** |
| FCD115HACXX:6:1208:14444:29719#TCTTATAT | 42 | 59 | 100 | chr27 | 100 | 0 | **1,721,521** | 1,721,480 |
| FCD115HACXX:6:1302:17328:82816#TCTTATAT | 30 | 1 | 30 | chr27 | 100 | 0 | 4,470,360 | **4,470,331** |
| FCD115HACXX:6:1302:17328:82816#TCTTATAT | 72 | 29 | 100 | chr27 | 100 | 0 | **1,721,521** | 1,721,450 |
| FCD115HACXX:6:1306:9829:150152#TCTTATAT | 53 | 1 | 53 | chr27 | 100 | 0 | 4,470,383 | **4,470,331** |
| FCD115HACXX:6:1306:9829:150152#TCTTATAT | 49 | 52 | 100 | chr27 | 100 | 0 | **1,721,521** | 1,721,473 |
| FCD115HACXX:6:2103:20960:179113#TCTTATAT | 58 | 1 | 58 | chr27 | 100 | 0 | 4,470,388 | **4,470,331** |
| FCD115HACXX:6:2103:20960:179113#TCTTATAT | 44 | 57 | 100 | chr27 | 100 | 0 | **1,721,521** | 1,721,478 |
| FCD115HACXX:6:2105:12382:134305#TCTTATAT | 50 | 1 | 50 | chr27 | 100 | 0 | 4,470,380 | **4,470,331** |
| FCD115HACXX:6:2105:12382:134305#TCTTATAT | 52 | 49 | 100 | chr27 | 100 | 0 | **1,721,521** | 1,721,470 |
| FCD115HACXX:6:2206:1471:101998#TCTTATAT | 27 | 1 | 27 | chr27 | 100 | 0 | 4,470,357 | **4,470,331** |
| FCD115HACXX:6:2206:1471:101998#TCTTATAT | 75 | 26 | 100 | chr27 | 100 | 0 | **1,721,521** | 1,721,447 |
| FCD115HACXX:6:2206:9435:81695#TCTTATAT | 71 | 1 | 71 | chr27 | 100 | 0 | 4,470,401 | **4,470,331** |
| FCD115HACXX:6:2206:9435:81695#TCTTATAT | 31 | 70 | 100 | chr27 | 100 | 0 | **1,721,521** | 1,721,491 |
| FCD115HACXX:6:2307:10019:103052#TCTTATAT | 65 | 1 | 65 | chr27 | 100 | 0 | 4,470,395 | **4,470,331** |
| FCD115HACXX:6:2307:10019:103052#TCTTATAT | 37 | 64 | 100 | chr27 | 100 | 0 | **1,721,521** | 1,721,485 |
| CNV3_CNV2 |  |  |  |  |  |  |  |  |
| FCD115HACXX:6:1201:20718:157711#TCTTATAT | 50 | 51 | 100 | chr27 | 100 | 0 | **4,503,417** | 4,503,368 |
| FCD115HACXX:6:1201:20718:157711#TCTTATAT | 48 | 1 | 48 | chr27 | 100 | 0 | 3,578,456 | **3,578,409** |
| FCD115HACXX:6:1206:3578:195950#TCTTATAT | 37 | 64 | 100 | chr27 | 100 | 0 | **4,503,417** | 4,503,381 |
| FCD115HACXX:6:1206:3578:195950#TCTTATAT | 61 | 1 | 61 | chr27 | 100 | 0 | 3,578,469 | **3,578,409** |
| FCD115HACXX:6:1304:8122:104325#TCTTATAT | 37 | 64 | 100 | chr27 | 100 | 0 | **4,503,417** | 4,503,381 |
| FCD115HACXX:6:1304:8122:104325#TCTTATAT | 61 | 1 | 61 | chr27 | 100 | 0 | 3,578,469 | **3,578,409** |
| FCD115HACXX:6:1306:18874:96478#TCTTATAT | 62 | 39 | 100 | chr27 | 100 | 0 | **4,503,417** | 4,503,356 |
| FCD115HACXX:6:1306:18874:96478#TCTTATAT | 36 | 1 | 36 | chr27 | 100 | 0 | 3,578,444 | **3,578,409** |
| FCD115HACXX:6:2101:5134:36660#TCTTATAT | 70 | 31 | 100 | chr27 | 100 | 0 | **4,503,417** | 4,503,348 |
| FCD115HACXX:6:2101:5134:36660#TCTTATAT | 28 | 1 | 28 | chr27 | 100 | 0 | 3,578,436 | **3,578,409** |
| FCD115HACXX:6:2201:19650:106663#TCTTATAT | 46 | 55 | 100 | chr27 | 100 | 0 | **4,503,417** | 4,503,372 |
| FCD115HACXX:6:2201:19650:106663#TCTTATAT | 52 | 1 | 52 | chr27 | 100 | 0 | 3,578,460 | **3,578,409** |
| FCD115HACXX:6:2306:10198:169736#TCTTATAT | 50 | 51 | 100 | chr27 | 100 | 0 | **4,503,417** | **4,503,368** |
| FCD115HACXX:6:2306:10198:169736#TCTTATAT | 48 | 1 | 48 | chr27 | 100 | 0 | 3,578,456 | **3,578,409** |
| FCD115HACXX:6:2308:14474:42004#TCTTATAT | 27 | 74 | 100 | chr27 | 100 | 0 | **4,503,417** | 4,503,391 |
| FCD115HACXX:6:2308:14474:42004#TCTTATAT | 71 | 1 | 71 | chr27 | 100 | 0 | 3,578,479 | **3,578,409** |
| CNV2_CNV1 |  |  |  |  |  |  |  |  |
| FCD115HACXX:6:2308:12762:50118#TCTTATAT | 48 | 43 | 90 | chr27 | 100 | 0 | **3,592,890** | 3,592,843 |
| FCD115HACXX:6:2308:12762:50118#TCTTATAT | 34 | 1 | 34 | chr27 | 100 | 0 | 1,702,302 | **1,702,269** |
| FCD115HACXX:6:1203:3219:150040#TCTTATAT | 41 | 60 | 100 | chr27 | 100 | 0 | **3,592,890** | 3,592,850 |
| FCD115HACXX:6:1203:3219:150040#TCTTATAT | 51 | 1 | 51 | chr27 | 100 | 0 | 1,702,319 | **1,702,269** |
| FCD115HACXX:6:1106:12798:72303#TCTTATAT | 39 | 62 | 100 | chr27 | 100 | 0 | **3,592,890** | 3,592,852 |
| FCD115HACXX:6:1106:12798:72303#TCTTATAT | 53 | 1 | 53 | chr27 | 100 | 0 | 1,702,321 | **1,702,269** |
| FCD115HACXX:6:1102:20380:73970#TCTTATAT | 48 | 46 | 93 | chr27 | 100 | 0 | **3,592,890** | 3,592,843 |
| FCD115HACXX:6:1102:20380:73970#TCTTATAT | 37 | 1 | 37 | chr27 | 97.3 | 1 | 1,702,305 | **1,702,269** |
| FCD115HACXX:6:1308:12454:178849#TCTTATAT | 48 | 39 | 86 | chr27 | 97.92 | 1 | **3,592,890** | 3,592,843 |
| FCD115HACXX:6:1308:12454:178849#TCTTATAT | 30 | 1 | 30 | chr27 | 100 | 0 | 1,702,298 | **1,702,269** |
| FCD115HACXX:6:1308:18668:112683#TCTTATAT | 48 | 38 | 85 | chr27 | 97.92 | 1 | **3,592,890** | 3,592,843 |
| FCD115HACXX:6:1308:18668:112683#TCTTATAT | 29 | 1 | 29 | chr27 | 100 | 0 | 1,702,297 | **1,702,269** |
| FCD115HACXX:6:1208:2626:196636#TCTTATAT | 43 | 58 | 100 | chr27 | 100 | 0 | **3,592,890** | 3,592,848 |
| FCD115HACXX:6:1208:2626:196636#TCTTATAT | 49 | 1 | 49 | chr27 | 100 | 0 | 1,702,317 | **1,702,269** |
| FCD115HACXX:6:2106:11055:165562#TCTTATAT | 30 | 71 | 100 | chr27 | 100 | 0 | **3,592,890** | 3,592,861 |
| FCD115HACXX:6:2106:11055:165562#TCTTATAT | 62 | 1 | 62 | chr27 | 100 | 0 | 1,702,330 | **1,702,269** |
| FCD115HACXX:6:2207:6685:65972#TCTTATAT | 45 | 56 | 100 | chr27 | 100 | 0 | **3,592,890** | 3,592,846 |
| FCD115HACXX:6:2207:6685:65972#TCTTATAT | 47 | 1 | 47 | chr27 | 100 | 0 | 1,702,315 | **1,702,269** |
| FCD115HACXX:6:2208:12326:137562#TCTTATAT | 54 | 47 | 100 | chr27 | 100 | 0 | **3,592,890** | 3,592,837 |
| FCD115HACXX:6:2208:12326:137562#TCTTATAT | 38 | 1 | 38 | chr27 | 100 | 0 | 1,702,306 | **1,702,269** |
