# Supplementary material for: A Complex Structural Variation on Chromosome 27 Leads to the Ectopic Expression of HOXB8 and the Muffs and Beard Phenotype in Chickens
Source: PLoS Genet. 2016 Jun 2;12(6):e1006071. doi: 10.1371/journal.pgen.1006071 (PMC4890787; doi:10.1371/journal.pgen.1006071)
Supplement: S4 Table — (DOCX) [file pgen.1006071.s011.docx]

**Table S4.** Product length of long-range PCRs.

| **Region** | **Length** |
| --- | --- |
| CNV1-copy1-5’ | 12 kb |
| CNV1-copy2-5’ | 12 kb |
| CNV1-copy1-3’ | 13 kb |
| CNV1-copy2-3’ | 11 kb |
| CNV2-copy1-5’ | 17 kb |
| CNV2-copy2-5’ | 19 kb |
| CNV2-copy1-3’ | 19 kb |
| CNV2-copy2-3’ | 19 kb |
| CNV3-copy1-5’ | 8 kb |
| CNV3-copy2-5’ | 7 kb |
| CNV3-copy1-3’ | 8 kb |
| CNV3-copy2-3’ | 6 kb |
