# Supplementary material for: A Complex Structural Variation on Chromosome 27 Leads to the Ectopic Expression of HOXB8 and the Muffs and Beard Phenotype in Chickens
Source: PLoS Genet. 2016 Jun 2;12(6):e1006071. doi: 10.1371/journal.pgen.1006071 (PMC4890787; doi:10.1371/journal.pgen.1006071)
Supplement: S5 Table — (DOCX) [file pgen.1006071.s012.docx]

**Table S5.** Primer information.

| **Purpose** | **Primer** | **Location (bp)** | **Sequence** |
| --- | --- | --- | --- |
| Breakpoint-searching | Breakpoint1_F | 1,698,809 | TAAATGCATAAACCCCCTTACG |
| Breakpoint-searching | Breakpoint2_R | 1,722,961 | ACCGAAGCAAAGAATAAATCCA |
| Breakpoint-searching | Breakpoint3_F | 4,469,853 | TCTCCCTCTCCCTTCACTGTTA |
| Breakpoint-searching | Breakpoint4_R | 4,506,190 | GAAGCAGACAGCCCTTGTCTAT |
| Breakpoint-searching | R1 | 1,701,097 | ACAGAACCCAAACAACTCCTGT |
| Breakpoint-searching & Diagnostic test | F1/CNV1_3_F | 1,719,940 | TTTGTTGTCTCCTTGCATCATT |
| Breakpoint-searching & Diagnostic test | R2/CNV1_3_R | 4,471,888 | CCATGTCAGCACAGTAACGATT |
| Breakpoint-searching & Diagnostic test | F2/CNV3_2_F | 4,503,286 | ATGTTGGTTGTGTGCCAAGTAG |
| Diagnostic test | CNV3_2_R | 3,578,757 | TTTCCCCTCGTCTGCTTTATTA |
| Diagnostic test | CNV2_1_F | 3,592,598 | ACAAGTCAGAGAGGCAATCGAC |
| Diagnostic test | CNV2_1_R | 1,702,365 | CCATACAGCCTCAGGTAAGGAC |
| Long-range PCR | CNV1_copy1_5’_F | 1,701,971 | CTCCTTGGCGTGTCATTATTAGGC |
| Long-range PCR | CNV1_copy2_5’_F | 3,592,595 | ATCACAAGTCAGAGAGGCAATCGAC |
| Long-range PCR | CNV1_copy1&2_5’_R | 1,713,018 | GCGTTCTGAGGGTTTCCATATTTC |
| Long-range PCR | CNV1_copy1&2_3’_F | 1,710,896 | AGGACTGAGGGACCTTGTGGAATAG |
| Long-range PCR | CNV1_copy1_3’_R | 1,723,953 | GTCAATTGGGTATGACGGGACATTA |
| Long-range PCR | CNV1_copy2_3’_R | 4,471,886 | CCATGTCAGCACAGTAACGATTTC |
| Long-range PCR | CNV2_copy1_5’_F | 4,469,702 | GGAAATCCAGCTCTGAACAAGTAA |
| Long-range PCR | CNV2_copy2_5’_F | 1,719,940 | TTTGTTGTCTCCTTGCATCATT |
| Long-range PCR | CNV2_copy1&2_5’_R | 4,486,423 | GTCCTAGGCTACATCCAAAGCTAA |
| Long-range PCR | CNV2_copy1_3’_F | 4,492,174 | CATTTGTTAATGCGCTCACTGGAG |
| Long-range PCR | CNV2_copy1_3’_R | 4,506,191 | ATGCAGACAGACACCTTCCAGAG |
| Long-range PCR | CNV2_copy2_3’_F | 4,491,695 | CATTGCTACTCTTCAGTCCAGCTA |
| Long-range PCR | CNV2_copy2_3’_R | 3,578,757 | TTTCCCCTCGTCTGCTTTATTA |
| Long-range PCR | CNV3_copy1_5’_F | 3,577,259 | GCTTGGGAAGCTCTTTATCGGT |
| Long-range PCR | CNV3_copy2_5’_F | 4,503,285 | TATGTTGGTTGTGTGCCAAGTAGA |
| Long-range PCR | CNV3_copy1&2_5’_R | 3,584,215 | GGAGCAGGGTAAAAGAATGATGTC |
| Long-range PCR | CNV3_copy1&2_3’_F | 3,585,842 | CTTTCTCTTTCTCCCTCGGTCTCAG |
| Long-range PCR | CNV3_copy1_3’_R | 3,593,635 | TTTCCAGAACCGTATTTTCGAGGA |
| Long-range PCR | CNV3_copy2_3’_R | 1,702,365 | CCATACAGCCTCAGGTAAGGAC |
| RACE | hoxb7_3’RACE_sp1 | 3,586,218 | AATATCAAGCCGCAAGTTCG |
| RACE | hoxb7_3’RACE_sp2 | 3,586,284 | CCTCCAACACCCAACGAG |
| RACE | hoxb8_3’RACE_sp1 | 3,581,819 | GGAGTTCTACCACGGAGCAT |
| RACE | hoxb8_3’RACE_sp2 | 3,581,851 | GCTCCCCTTACCAACAGAATC |
| RACE | smarcd2_3’RACE_sp1 | 1,709,947 | GAGGAAGATGGCTGACAAGG |
| RACE | smarcd2_3’RACE_sp2 | 1,709,327 | CGGAAGAGGATGGAGATTCA |
| RACE | smarce1_3’RACE_sp1 | 4,492,125 | TCACGGCTTCCTCTGGTATT |
| RACE | smarce1_3’RACE_sp2 | 4,490,885 | TTGGCAAGATTATTGGAGGAAT |
| RACE | krt222_3’RACE_sp1 | 4,504,270 | CTGTGGAAAAGGGTTTGGAG |
| RACE | krt222_3’RACE_sp2 | 4,504,198 | AGGCGGAGATTGAATGCTTA |
| RACE | ccr7_3’RACE_sp1 | 4,471,030 | GTCCGTGATTTCCGTGCT |
| RACE | ccr7_3’RACE_sp2 | 4,470,963 | GAAATGGGCTGGTGATGC |
| RACE | psmc5_3’RACE_sp1 | 1,701,706 | AGCAGATGGAGATGGACGAT |
| RACE | psmc5_3’RACE_sp2 | 1,702,543 | TATGTGGGTGAGGTGGTGAG |
| RACE | hoxb7_5’RACE_R1 | 3,589,277 | TCTCCTCCTCGTCAGGTAGC |
| RACE | hoxb7_5’RACE_R2 | 3,589,194 | GTTTCCTATCCGTGCCTGTG |
| RACE | hoxb7_5’RACE_R3 | 3,586,516 | GCAAATCTGAATCCCTCTGG |
| RACE | hoxb8_5’RACE_R1 | 3,583,153 | CTTCTGTGCTTCCCCTTCCT |
| RACE | hoxb8_5’RACE_R2 | 3,583,076 | GCTGCTGGGAAACTTGTCTT |
| RACE | hoxb8_5’RACE_R3 | 3,582,909 | TCTGGTAGCGGCTGTAGGTT |
| RACE | smarcd2_5’RACE_R1 | 1,706,282 | ACCTTGGCGAAGATGTGTCT |
| RACE | smarcd2_5’RACE_R2 | 1,706,781 | CTCCTGCTGGTTGGTGGT |
| RACE | smarcd2_5’RACE_R3 | 1,707,218 | GCATACGGACACAGTTGAAGAT |
| RACE | smarce1_5’RACE_R1 | 4,485,183 | GTGCTCTCTTCTGGGTGAGG |
| RACE | smarce1_5’RACE_R2 | 4,486,193 | TTCCATATCCACTTCCACCTTC |
| RACE | smarce1_5’RACE_R3 | 4,487,429 | ACCACACTTTCGCTCAGGAT |
| RACE | krt222_5’RACE_R1 | 4,501,277 | ATGACCAGCCTGACCTCAAT |
| RACE | krt222_5’RACE_R2 | 4,501,392 | GTGGGTTGTCCTTGAAGAAAGA |
| RACE | krt222_5’RACE_R3 | 4,502,766 | CTTCTAGGATTGCTTGTTTTGTCA |
| RACE | ccr7_5’RACE_R1 | 4,470,262 | GCAGGCTAGAGTGTAGGTCACA |
| RACE | ccr7_5’RACE_R2 | 4,470,384 | AGACGACGAAGACGATGACC |
| RACE | ccr7_5’RACE_R3 | 4,470,492 | TGACCACCAGGGGAACTAAG |
| RACE | psmc5_5’RACE_R1 | 1,704,468 | CCATTTCAAAGTCCTCCTGTGT |
| RACE | psmc5_5’RACE_R2 | 1,704,215 | CCGAGAGTGGATTTTGAGGA |
| RACE | psmc5_5’RACE_R3 | 1,703,745 | CCTCACTGTCCCCACCAG |
| RACE | 3’RACE_Oligo dT |  | CAAGCTGGTCCTGGACATCGAGATCACCACCTACCGCAAGCTTTTTTTTTTTTTTT |
| RACE | 3’RACE_L1 |  | CAAGCTGGTCCTGGACATC |
| RACE | 3’RACE_L2 |  | GATCACCACCTACCGCAAG |
| QPCR | Hoxb7_F | 3,589,247 | TGGAGCTGGAGAAGGAGTTC |
| QPCR | Hoxb7_R | 3,589,311 | CGTGAGACACAGAGCATGG |
| QPCR | HoxB8_F | 3,583,010 | TGACAGAAAGGCAGGTCAAA |
| QPCR | HoxB8_R | 3,583,076 | GCTGCTGGGAAACTTGTCTT |
| QPCR | SMARCE1_F | 4,487,350 | CCTTAATGGTTCACCAGCGTA |
| QPCR | SMARCE1_R | 4,486,200 | TCCACTTCCACCTTCAAACTG |
| QPCR | SMARCD2_F | 1,707,535 | TACATCAACTGCAACCGCTACT |
| QPCR | SMARCD2_R | 1,707,535 | GCTAACTTCATGGGGATCTCAG |
| QPCR | PSMC5_F | 1,703,812 | GCCACCAAGAACATCAAGGT |
| QPCR | PSMC5_R | 1,704,215 | CCGAGAGTGGATTTTGAGGA |
| QPCR | CCR7_F | 4,472,853 | CTGAGGGTCACCATTGCTTT |
| QPCR | CCR7_R | 4,471,043 | CGGACCTCCTTCTTCTCACA |
| QPCR | KRT222_F | 4,503,005 | CCTCAACACAACTGCCTTCA |
| QPCR | KRT222_R | 4,502,733 | CATGAGCTTCAGCACTTTGC |
| QPCR | MSX2_F | 9,129,344 | CTCCTCCAAGACACCTGAGC |
| QPCR | MSX2_R | 9,132,942 | TACTGCTTCTGGCGGAACTT |
| QPCR | GAPDH_F | 76,434,730 | CGATCTGAACTACATGGTTTACATGTT |
| QPCR | GAPDH_R | 76,435,070 | CCCGTTCTCAGCCTTGACA |
